# Clinical, Immunological, and Molecular Features of Severe Combined Immune Deficiency: A Multi-Institutional Experience From India

**DOI:** 10.3389/fimmu.2020.619146

**Published:** 2021-02-08

**Authors:** Pandiarajan Vignesh, Amit Rawat, Rajni Kumrah, Ankita Singh, Anjani Gummadi, Madhubala Sharma, Anit Kaur, Johnson Nameirakpam, Ankur Jindal, Deepti Suri, Anju Gupta, Alka Khadwal, Biman Saikia, Ranjana Walker Minz, Kaushal Sharma, Mukesh Desai, Prasad Taur, Vijaya Gowri, Ambreen Pandrowala, Aparna Dalvi, Neha Jodhawat, Priyanka Kambli, Manisha Rajan Madkaikar, Sagar Bhattad, Stalin Ramprakash, Raghuram CP, Ananthvikas Jayaram, Meena Sivasankaran, Deenadayalan Munirathnam, Sarath Balaji, Aruna Rajendran, Amita Aggarwal, Komal Singh, Fouzia Na, Biju George, Ankit Mehta, Harsha Prasada Lashkari, Ramya Uppuluri, Revathi Raj, Sandip Bartakke, Kirti Gupta, Sreejesh Sreedharanunni, Yumi Ogura, Tamaki Kato, Kohsuke Imai, Koon Wing Chan, Daniel Leung, Osamu Ohara, Shigeaki Nonoyama, Michael Hershfield, Yu-Lung Lau, Surjit Singh

**Affiliations:** ^1^ Allergy Immunology Unit, Department of Pediatrics, Advanced Pediatrics Centre, Post Graduate Institute of Medical Education and Research, Chandigarh, India; ^2^ Bone Marrow Transplantation Unit, Department of Internal Medicine, Post Graduate Institute of Medical Education and Research, Chandigarh, India; ^3^ Department of Immunopathology, Post Graduate Institute of Medical Education and Research, Chandigarh, India; ^4^ Department of Immunology, Bai Jerbai Wadia Hospital for Children, Mumbai, India; ^5^ Bone Marrow Transplantation Unit, Bai Jerbai Wadia Hospital for Children, Mumbai, India; ^6^ ICMR-National Institute of Immunohematology, Mumbai, India; ^7^ Pediatric Immunology and Rheumatology, Aster CMI hospital, Bengaluru, India; ^8^ Pediatric Hemat-oncology and BMT Unit, Aster CMI Hospital, Bengaluru, India; ^9^ Anand Neuberg Diagnostic and Research Centre, Bengaluru, India; ^10^ Kanchi Kamakoti Child Trust Hospitals for Children, Chennai, India; ^11^ Institute of Child Health, Madras Medical College, Chennai, India; ^12^ Sanjay Gandhi Postgraduate Institute of Medical Sciences, Lucknow, India; ^13^ Christian Medical College, Vellore, India; ^14^ Zydus Hospitals, Ahmedabad, India; ^15^ Department of Pediatrics, Kasturba Medical College, Mangalore, India; ^16^ Apollo Children’s Hospital, Chennai, India; ^17^ Aditya Birla Memorial Hospital, Pune, India; ^18^ Department of Histopathology, Post Graduate Institute of Medical Education and Research, Chandigarh, India; ^19^ Department of Hematology, Post Graduate Institute of Medical Education and Research, Chandigarh, India; ^20^ Department of Pediatrics, National Defense Medical College, Saitama, Japan; ^21^ Department of Community Pediatrics, Perinatal and Maternal Medicine, Tokyo Medical and Dental University, Tokyo, Japan; ^22^ Department of Paediatrics and Adolescent Medicine, The University of Hong Kong, Hong Kong, Hong Kong; ^23^ Kazusa DNA Research Institute, Chiba, Japan; ^24^ Duke University Medical Center, Durham, NC, United States

**Keywords:** severe combined immune deficiency, India, hematopoietic stem cell transplantation, newborn screening, BCG

## Abstract

**Background:**

Severe Combined Immune Deficiency (SCID) is an inherited defect in lymphocyte development and function that results in life-threatening opportunistic infections in early infancy. Data on SCID from developing countries are scarce.

**Objective:**

To describe clinical and laboratory features of SCID diagnosed at immunology centers across India.

**Methods:**

A detailed case proforma in an Excel format was prepared by one of the authors (PV) and was sent to centers in India that care for patients with primary immunodeficiency diseases. We collated clinical, laboratory, and molecular details of patients with clinical profile suggestive of SCID and their outcomes. Twelve (12) centers provided necessary details which were then compiled and analyzed. Diagnosis of SCID/combined immune deficiency (CID) was based on 2018 European Society for Immunodeficiencies working definition for SCID.

**Results:**

We obtained data on 277 children; 254 were categorized as SCID and 23 as CID. Male-female ratio was 196:81. Median (inter-quartile range) age of onset of clinical symptoms and diagnosis was 2.5 months (1, 5) and 5 months (3.5, 8), respectively. Molecular diagnosis was obtained in 162 patients - *IL2RG* (36), *RAG1* (26), *ADA* (19), *RAG2* (17), *JAK3* (15), *DCLRE1C* (13), *IL7RA* (9), *PNP* (3), *RFXAP* (3), *CIITA* (2), *RFXANK* (2), *NHEJ1* (2), *CD3E* (2), *CD3D* (2), *RFX5* (2), *ZAP70* (2), *STK4* (1), *CORO1A* (1), *STIM1* (1), *PRKDC* (1), *AK2* (1), *DOCK2* (1), and *SP100* (1). Only 23 children (8.3%) received hematopoietic stem cell transplantation (HSCT). Of these, 11 are doing well post-HSCT. Mortality was recorded in 210 children (75.8%).

**Conclusion:**

We document an exponential rise in number of cases diagnosed to have SCID over the last 10 years, probably as a result of increasing awareness and improvement in diagnostic facilities at various centers in India. We suspect that these numbers are just the tip of the iceberg. Majority of patients with SCID in India are probably not being recognized and diagnosed at present. Newborn screening for SCID is the need of the hour. Easy access to pediatric HSCT services would ensure that these patients are offered HSCT at an early age.

## Introduction

Severe Combined Immune Deficiency (SCID) is an inborn error of immunity characterized by defect in T lymphocyte development and function. Children with SCID often develop life-threatening opportunistic fungal, bacterial, or viral infections in early infancy. SCID is considered a medical emergency and affected children often succumb to severe infections if diagnosis and definitive treatment are delayed. The estimated incidence of SCID is 1 in 50,000 to 100,000 live births (1). Recent data also suggest an incidence of SCID as high as 1 in 3,000 live births in countries with high consanguinity rates (2). However, due to lack of awareness and diagnostic facilities in developing countries, diagnosis is often missed. Hematopoietic stem cell transplantation (HSCT) is the definitive management for SCID. Early diagnosis and management are essential for successful outcomes. Several countries such as United States of America, Israel, Germany, Switzerland, Sweden, Norway, Iceland, New Zealand, and Taiwan have initiated newborn screening for SCID based on quantification of T-cell receptor excision circles (TRECs) to facilitate early diagnosis (3).

Opportunistic infections in SCID are recurrent, typically start in early infancy, and result in failure to thrive. Common infection patterns seen in SCID include oral thrush, disseminated BCGosis, disseminated cytomegalovirus, and life-threatening bacterial and fungal infections. Non-infective manifestations of SCID include Omenn syndrome (OS), graft versus host reaction, autoimmunity, and hemophagocytic lymphohistiocytosis (4). CD3+ T lymphocyte numbers are usually decreased in SCID (T-). However, in cases of maternal T-cell engraftment or OS, CD3+ T cell numbers can be normal or increased. The expanded T cells are autoreactive in OS, whereas, they are alloreactive in cases with transplacental-acquired maternal T-cell engraftment. T lymphocyte function and naïve T cell numbers are reduced in such cases. T- SCID can be classified based on presence or absence of B lymphocytes and natural killer cells as T-B-NK+, T-B-NK-, T-B+NK-, and T-B+NK+. Combined immunodeficiencies (CID) are also characterized by presence of opportunistic infections and immune dysregulation; however, the age of onset is little older and have a milder immunodeficiency compared to SCID (5).

Until date, 58 different monogenic defects have been identified to result in immunodeficiencies affecting both cellular and humoral immunity and 18 amongst these are known to result in SCID (5). Molecular defects in SCID can be broadly classified as abnormalities in VDJ recombination (*RAG1, RAG2*, *DCLRE1C, NHEJ1, LIG4, PRKDC*), abnormalities of cytokine signaling (*IL2RG, JAK3, IL7RA*), toxic metabolite accumulation (*ADA, PNP*), defective survival of hematopoietic precursors (*AK2, RAC2*), abnormalities of T-cell receptor and signaling (*PTPRC, CD3D, CD3E, CD3Z, LAT*), and abnormalities of actin cytoskeleton (*CORO1A*). While X-linked SCID due to defect in *IL2RG* is considered to be the commonest form of SCID in the US, Canada, and Europe, autosomal recessive form of SCID due to defects in *RAG1/2* are the commonest forms of SCID in countries where consanguinity rates are high (6–8). However, after initiation of newborn screening program, defects in *RAG1/RAG2* are now increasingly being identified even in countries like US and Canada where consanguinity rates are low (9).

Reports of clinical data and outcomes of SCID from developing nations are scarce. Being a tropical nation with universal coverage of BCG vaccination in newborns, microbiological pattern of infections in SCID in India is expected to be different from other cohorts. Molecular spectrum is also expected to be different considering high rates of consanguinity and endogamous marriages in India (6–8). A recent cohort of 57 patients from Mumbai, India showed a high incidence of autosomal recessive forms of SCID with *RAG1/2* defects being the commonest (7). We aim to describe the clinical, immunological, and molecular features of children with SCID in this large multicentric cohort from India.

## Methods

A detailed case proforma in an Excel format was prepared by one of the authors (PV) and was sent to centers that are recognized as Foundation for Primary Immunodeficiency Diseases (FPID) centers for care of primary immunodeficiencies in India. The format was also sent to tertiary-care centers that manage patients with primary immunodeficiency diseases (PIDs). Information on clinical, laboratory, and molecular details of patients with SCID and their outcomes was sought and collated. Twelve (12) centers provided details of 319 patients that were then compiled and analyzed. Fifteen (15) patients from 2 other centers with either flow-cytometry or mutation-proven SCID are not included in final analysis as data were incomplete. Twenty-three (23) children did not fulfil the criteria for clinical definition for SCID and were not included for analysis. Duplicate entries (n=4) were also noted and excluded.

Data of 277 children who had a clinical profile suggestive of SCID were taken for final analysis (**Supplementary Table 1**). Children were categorized as SCID/OS/CID/atypical SCID as per the European Society for Immunodeficiencies (ESID) working definition (10). Three (3) patients were classified as possible SCID as they did not fulfil the complete ESID definition, however, the treating team had a high index of suspicion based on clinical and immunological features (**Table 1**).

**Table 1:** Clinical and immunological features of children with clinical features suggestive of SCID in our cohort.

S No	Age/Sex	Clinical features	Organisms isolated	Absolute lymphocyte count	Immunoglobulin profile	Lymphocyte subsets	Molecular defect	ESID Working Definition
Pt. 1	8 months/male	Recurrent episodes of diarrhea, failure to thrive, pneumonia, meningitis	Stool: *Clostridium difficile* toxin assay positive	2.260	IgG <1.64 g/L IgA <0.36 g/L IgM- 0.25 g/L	CD3- 0.3% (No: 6-7) CD19- 66% (No: 1492) CD56- 30% (No: 675)	Not done	SCID
Pt. 2	5 months/male	Recurrent episodes of pneumonia, diarrhea, failure to thrive, elder male sibling expired at 6 months due to severe infections	Blood culture: *Alcaligens faecalis*	0.410	IgG <2.26 g/L IgA <0.1 g/L IgM<0.2 g/L	CD3- 0.15% (No: 0-1) CD19- 0% (No: 0) CD56- 84% (No: 345)	Not done	SCID
Pt. 3	6.5 months/male	Recurrent episodes of pneumonia, meningitis, hepatosplenomegaly, pancytopenia, transaminitis (HLH), 3 elder male siblings died at early infancy due to recurrent infections	Blood culture: *Pseudomonas aeruginosa* Disseminated BCGosis and angioinvasive aspergillosis in lungs in autopsy	0.940	IgG<2.99 g/L IgA- 0.49 g/L IgM- 0.88 g/L	CD3- 0% CD19- 86% (808) CD56- 0.3%	*IL2RG*	SCID
Pt. 4	5 months/male	2 episodes of pneumonia, recurrent diarrhea, umbilical sepsis, failure to thrive, 3 elder male siblings died at early infancy due to recurrent infections	N.A.	2.050	IgG- 2.64 g/L IgA <0.46 g/L IgM- 0.18 g/L	CD3- 0% CD19- 96.1% (1968) CD56- 0%	*IL2RG*	SCID
Pt. 5	3 months/male	Erythroderma, generalized adenopathy, diarrhea, lymphocytosis, eosinophilia (Omenn syndrome), failure to thrive, elder male sibling died due to eczema and pneumonia at 3^rd^ month	N.A.	18.540	N.A.	CD3- 70.94% (13,124) CD19- 0.1% CD56- 7% (1,295)	*RAG2*	Omenn syndrome
Pt. 6	6 months/male	Persistent pneumonia, oral thrush, 7 maternal uncles died at early infancy due to recurrent infections	N.A.	2.322	IgG- 0.65 g/L IgA- 0.22 g/L IgM- 0.24 g/L	CD3- 0% CD19- 96.75% (2,245) CD56- 3.2% (74)	*IL2RG*	SCID
Pt. 7	3 months/male	Recurrent episodes of pneumonia, diarrhea, meningitis, generalized erythroderma (incomplete Omenn), elder male sibling died at early infancy due to rash and pneumonia	N.A.	1.566	IgG- 2.14 g/L IgM- 0.24 g/L	CD3- 74.79% (1167) CD19- 0.27% (42) CD56- 23% (360) CD3+45RA+ 45RO-: 18.65% compared to 82% in control	Not done	Omenn syndrome
Pt. 8	10 months/male	Recurrent episodes of diarrhea, pneumonia, otitis media, failure to thrive, BCG site ulceration, hepatosplenomegaly, generalized adenopathy, erythroderma, eosinophilia (Omenn syndrome), 5 maternal uncles died at early infancy due to recurrent infections	Disseminated BCGosis, disseminated *Mycobacterium avium*, disseminated CMV, and Aspergillus pneumonia in autopsy	3.600	IgG- 1.04 g/L IgA- 0.07 g/L IgM- 0.31 g/L IgE- 700 U/L (Normal: 0.-6 U/L)	CD3- 95.79% (3,448) CD19- 0.2% (7) CD56- 1% (36)	*IL2RG*	Omenn syndrome
Pt. 9	2 months/female	Recurrent episodes of oral thrush, failure to thrive, 1 elder male sibling expired due to sepsis in early infancy	N.A.	0.648	IgG- 2.72 g/L IgA- 0.09 g/L IgM- 0.41 g/L	CD3- 1.1% (7) CD19- 0.2% (1) CD56- 93.6% (607)	*DCLRE1C*	SCID
Pt. 10	3 months/male	Recurrent episodes of pneumonia, diarrhea, rickets, nephrocalcinosis, distal renal tubular acidosis, oral thrush, failure to thrive	N.A.	0.896	IgG- 0.88 g/L IgA <0.06 g/L IgM- 0.19 g/L	CD3- 1.1% (10) CD19- 92.2% (8,26) CD56- 6.4% (57)	*IL7RA*	SCID
Pt. 11	6 months/male	Pustulosis, hepatosplenomegaly, BCG site ulceration, transfusion-associated GVHD, elder male sibling died at 5 months due to pneumonia	Disseminated BCGosis, Blood culture: *Enterobacter* sp.	1.462	N.A.	CD3- 1.25% (183) CD19- 95% (1389) CD56- 0.45% (6-7)	No gene variants found in *IL2RG, JAK3, RAG1, RAG2*	SCID
Pt. 12	4 months/male	Recurrent episodes of pneumonia, diarrhea, failure to thrive, meningitis, oral thrush, hepatosplenomegaly, rash, eosinophilia (Omenn phenotype), one elder female sibling expired in early infancy	*Pneumocystis jirovecii* pneumonia, disseminated CMV in autopsy	4.176	IgG- 2.06 g/L IgA- 0.08 g/L IgM- 0.41 g/L	CD3- 71.6% (2,993) CD19- 1.0% (42) CD56- 12% (504) CD3+45RO-45RA+: 24% as compared to 82% in control	Not done	Omenn syndrome
Pt. 13	6 months/male	Persistent pneumonia, diarrhea, oral thrush, erythematous rash, hepatosplenomegaly (incomplete Omenn), nephrotic range proteinuria, two elder siblings (one male and other female) expired in early infancy	Blood culture: *Acinetobacter* sp.; Pneumonia and meningitis due to *Aspergillus* sp. and ventriculitis due to CMV in autopsy	1.404	IgG- 2.46 g/L IgA- 0.37 g/L IgM- 1.38 g/L	CD3- 93.7% (1,312) CD19- 0.2% (3) CD56- 5.6% (78)	Not done	Omenn syndrome
Pt. 14	7 months/male	Recurrent pneumonia, failure to thrive, oral thrush	Endotracheal aspirate: *Klebsiella* sp.; RSV pneumonia and disseminated CMV in autopsy	0.156	IgG- 7.86 g/L IgA- 0.61 g/L IgM <0.11 g/L	CD3- 45.7% (72) CD19- 1.6% (2-3) CD56- 21.7% (34)	*PNP*	SCID
Pt. 15	6 months/male	Recurrent episodes of pneumonia, failure to thrive, 5 elder siblings died at early infancy	N.A.	1.391	IgG <0.93 g/L IgA <0.16 g/L IgM <0.11 g/L	CD3- 0% CD19- 0% CD56- 92.2% (1,291)	*RAG2*	SCID
Pt. 16	6 months/female	Recurrent pneumonia, diarrhea, failure to thrive, hepatosplenomegaly	Blood culture: *Candida* sp.	0.785	IgG- 6.33 g/L IgA- 0.07 g/L IgM <0.11 g/L	CD3- 0.2% (1-2) CD19- 38.9% (312) CD56- 52.2% (407)	*IL7RA*	SCID
Pt. 17	4 months/male	Recurrent pneumonia, pus discharging sinuses in neck, generalized rash (incomplete Omenn), 3 elder siblings (one female and 2 male) died in early infancy	CMV PCR+, Blood culture: *Enterococcus cloacae*	1.800	IgG <0.95 g/L IgA <0.17 g/L IgM- 0.12 g/L	CD3- 83.3% (1,499) CD19- 0.2% (3-4) CD56- 14.3% (257) CD3+45RO-45RA+: 27.5% compared to 82% in control	*RAG2*	Omenn syndrome
Pt. 18	9 months/male	2 episodes of pneumonia, failure to thrive, meningoencephalitis and hydrocephalus, MRI Brain: multiple tuberculomas noted over parietal and occipital area, 2 elder male siblings expired in early infancy	N.A.	0.612	IgG <0.95 g/L IgA <0.17 g/L IgM <0.15 g/L	CD3- 4.2% CD19- 0.2% CD56- 85%	*RAG1*	SCID
Pt. 19	2 months/male	Recurrent episodes of pneumonia, failure to thrive	N.A.	0.655	IgG- 2.02 g/L IgA <0.16 g/L IgM <0.11 g/L	CD3- 0% CD19- 0.13% (1) CD56- 72% (468)	*RAG1*	SCID
Pt. 20	5 months/male	Generalized rash, alopecia, loose stools (incomplete Omenn), failure to thrive, meningitis	N.A.	1.372	IgA <0.17 g/L IgM <0.12 g/L	CD3- 69.6% (954) CD19- 0.15% (2) CD56- 10.7% (147)	*DCLRE1C*	Omenn syndrome
Pt. 21	5 months/male	Younger sibling of Pt. 15, recurrent episodes of pneumonia, diarrhea, failure to thrive	CMV DNA PCR positive	0.480	IgG <0.94 g/L IgA- 0.18 g/L IgM <0.12 g/L	CD3- 21.7% (109) CD19- 1% (5) CD56- 86% (430)	*RAG2*	SCID
Pt. 22	1.5 months/male	Persistent pneumonia, diarrhea, elder female sibling expired at early infancy	Blood culture: *Candida* sp.	0.328	IgG <2.02 g/L IgA <0.17 g/L	CD3- 75% (248) CD19- 8.3% (27) CD56- 7.1% (23)	*ADA*	SCID
Pt. 23	5 months/male	Oral thrush, pneumonia, meningitis, one elder female sibling expired due to anemia and pneumonia in early infancy	Disseminated CMV and early invasive pulmonary aspergillosis in autopsy	0.788	IgG- 2.49 g/L	CD3- 0.79% (6) CD19- 1.02% (8) CD56- 92.7% (744)	*RAG1*	SCID
Pt. 24	2 years/male	Recurrent pneumonia, diarrhea, otitis media, failure to thrive, esophageal candidiasis	N.A.	8.567	IgG- 5.19 g/L IgA <0.17 g/L IgM- 0.85 g/L	CD3- 25.76% (2,236) CD3+CD4+- 33.5% (737) CD3+CD8+- 50.2% (1,104) CD19- 51.95% (4,451) CD56- 11.6% (994) CD3+45RA+45RO-: 31.7% compared to 74% in control	No gene variants found	CID
Pt. 25	4 months/male	Younger sibling of pt. 8, recurrent episodes of pneumonia and diarrhea, failure to thrive	N.A.	5.280	IgG <2.05 g/L IgM <0.25 g/L	CD3- 0.23% (12) CD19- 94.6% (4,995) CD56- 0.47% (25)	*IL2RG*	SCID
Pt. 26	10 months/male	Recurrent episodes of pneumonia, failure to thrive	N.A.	0.378	N.A.	CD3- 2.7% CD19- 2.15% CD56- 85.2%	*DCLRE1C*	SCID
Pt. 27	2.5 months/female	Recurrent pneumonia, otitis media, oral thrush, diarrhea, erythroderma, hepatosplenomegaly, eosinophilia (incomplete Omenn syndrome), elder female sibling expired in early infancy	N.A.	1.650	IgG- 1.23 g/L IgA <0.17 g/L IgM <0.25 g/L	CD3- 7.67% (127) CD19- 0.69% (11) CD56- 82.7% (1,365) CD3+45RA+45RO-: 6.42% compared to 72% of control	*RAG2*	SCID/Omenn syndrome
Pt. 28	5 months/male	Recurrent episodes of pneumonia, failure to thrive	BAL: *Pseudomonas* sp.; Blood culture: *Candida* sp.	0.360	IgG <2.05 g/L IgA- 0.07 g/L IgM <0.05 g/L	CD3- 2.3% (8) CD19- 3.8% (14) CD56- 92.2% (332)	*RAG1*	SCID
Pt. 29	8 months/female	Persistent pneumonia, recurrent episodes of diarrhea, failure to thrive, chorioretinitis, hepatosplenomegaly	Disseminated CMV; Blood culture: *Acinetobacter baumanii*	1.316	IgG- 4.17 g/L IgA- 0.22 g/L	CD3- 11.3% (149) CD19- 69.8% (921) CD56- 1.75% (23)	*JAK3*	SCID
Pt. 30	1.5 months/male	Recurrent episodes of pneumonia, diarrhea, failure to thrive, elder male sibling died in early infancy	Blood culture: *Acinetobacter baumanii*	0.204	IgG- 1.96 g/L IgM <0.25 g/L	CD3- 54% (108) CD19- 24% (48) CD56- 20% (40)	*ADA*	SCID
Pt. 31	4 years/male	Recurrent episodes of pneumonia since early infancy, failure to thrive	N.A.	0.116	IgG- 4.73 g/L IgA- 1.05 g/L IgM- 1.12 g/L	CD3- 64.8% (78) CD19- 4% (5) CD56- 7% (8-9)	*ADA*	Atypical SCID
Pt. 32	9 months/male	Recurrent episodes of pneumonia, diarrhea, failure to thrive	N.A.	0.154	IgG- 2.27 g/L IgA- 0.27 g/L IgM <0.25 g/L	CD3- 44.4% (67) CD19- 38.5% (58) CD56- 5.7% (9)	Not done	SCID
Pt. 33	2 months/female	Recurrent episodes of pneumonia, diarrhea, failure to thrive	N.A.	0.977	IgG- 2.45 g/L IgA- 0.23 g/L IgM- 0.29 g/L	CD3- 32% (314) CD19- 57% (559) CD56- 1.2% (12)	Not done	SCID
Pt. 34	5 months/male	Recurrent diarrhea, failure to thrive, BCG site ulceration, pneumonia, erythroderma, eosinophilia, alopecia (Omenn syndrome)	Blood culture: Enterococcus sp.; *Pneumocystis jirovecii* pneumonia and disseminated BCGosis in autopsy	2.498	IgG <2.05 g/L IgM- 0.34 g/L IgE- 369 kU/L (up to 7.3)	CD3- 78.01% (1,950) CD19- 4.44% (110) CD56- 13.12% (325) CD3+45RA+RO-: 2.26% compared to 83.7% in control CD3+HLA-DR+: 86.25% compared to 8.6% in control	No gene variants found	Omenn syndrome
Pt. 35	6 months/male	Recurrent pneumonia, failure to thrive, elder male sibling expired in early infancy due to pneumonia	N.A.	0.868	N.A.	CD3- 3% (26) CD19- 94% (818) CD56- 0.4% (3)	*IL2RG*	SCID
Pt. 36	1.5 months/female	Anasarca, nephrotic range proteinuria, pneumonia, failure to thrive, erythematous rash (incomplete Omenn), elder male sibling expired in early infancy	N.A.	0.722	IgG- 8.29 g/L IgA- 0.75 g/L	CD3- 89% CD3+CD4+- 8% CD3+CD8+- 85.1% CD19- 0.3% CD56- 0.8% CD3+45RA+45RO-: 30% compared to 90% in control CD3+45RA-45RO+: 79.14% compared to 19.24% in control CD3+HLA-DR+: 90.13% compared to 12.7% in control	*ADA*	Omenn syndrome
Pt. 37	5 months/female	Recurrent pneumonia, diarrhea, failure to thrive, BCG site ulceration	N.A.	0.861	IgG <2.0 g/L IgA <0.17 g/L	CD3- 34% (292) CD3+CD4+- 29.7% (89) CD3+CD8+- 55.3% (165) CD19- 45% (387) CD56- 12.1% (103)	Not done	SCID
Pt. 38	5 months/male	Recurrent pneumonia, diarrhea, failure to thrive	N.A.	0.140	N.A.	CD3- 9.6% (13) CD19- 8.7% (12) CD56- 80% (112)	*RAG1*	SCID
Pt. 39	2 months/male	Recurrent episodes of diarrhea, failure to thrive, sacral abscess, 2 elder siblings died in early infancy due to repeated infections	Blood culture: Staphylococcus aureus; Disseminated CMV in autopsy	0.06	N.A.	CD3- 50% (30) CD19- 7.7% (4-5) CD56- 34.6% (21)	*ADA*	SCID
Pt. 40	2.5 months/male	Recurrent pneumonia, otitis media, failure to thrive, 6 maternal uncles and 2 elder male siblings died at early infancy due to repeated infections	N.A.	1.406	N.A.	CD3- 0.07% (01) CD19- 91.5% (1,598) CD56- 1.8% (33)	Not done	SCID
Pt. 41	4 years/female	Eczematoid eruptions and chronic otitis media since early infancy, autoimmune hemolytic anemia, generalized adenopathy	N.A.	1.922	IgG- 21.56 g/L IgA- 4.77 g/L IgM- 0.57 g/L IgE- 933 U/L (Normal: up to 60)	CD3- 24.79% (912) CD3+CD4+- 21.2% (193) CD3+CD8+- 55% (500) CD19- 42.3% (812) CD56- 2.7% (58) CD3+45RA+RO-: 45% compared to 76% in control CD3+CD4+45RA+RO-: 14.9% compared to 67% in control CD3+CD8+45RA+45RO-: 35.8% compared to 72% in control	*STK4*	CID
Pt. 42	4 months/male	Recurrent pneumonia, diarrhea, failure to thrive, oral thrush, 1 maternal uncle died at 2 years due to repeated infections	Blood culture: *Moraxella* sp.	1.302	N.A.	CD3- 1.3% (17) CD19- 85.16% (1,109) CD56- 2.9% (37)	Not done	SCID
Pt. 43	2.5 months/male	Chronic diarrhea, failure to thrive, esophageal candidiasis, maternal cousin (male) expired at early infancy due to pneumonia	N.A.	0.415	N.A.	CD3- 3.8% (15) CD19- 84% (336) CD56- 3% (12)	*IL2RG*	SCID
Pt. 44	3 months/male	Recurrent pneumonia, diarrhea, failure to thrive, erythroderma, eosinophilia, hepatosplenomegaly (maternal T cell engraftment), 1 maternal uncle died at early infancy due to pneumonia	Blood culture: *Weisella confusa*	7.457	N.A.	CD3- 15.9% (1,192) CD19- 76.4% (5,692) CD56- 1.9% (142) CD3+45RA+RO-: 5.43% compared to 59% in control CD3+45RA-45RO+: 96.9% compared to 60% in control CD3+HLA-DR+: 83.5% compared to 15.7% in control	*IL2RG*	Atypical SCID
Pt. 45	4 months/male	Recurrent pneumonia, diarrhea, failure to thrive, oral thrush, BCG site ulceration	N.A.	2.831	IgG <0.87 g/L IgA <0.16 g/L	CD3- 0.2% (5-6) CD19- 97.7% (2,765) CD56- 0.48% (13-14)	No gene variants found	SCID
Pt. 46	5 months/male	Recurrent fever, BCG site ulceration, hepatosplenomegaly, oral thrush	Disseminated BCGosis	2.086	IgG <1.46 g/L	CD3- 0.6% (13) CD19- 97.8% (2,044) CD56- 0.2% (4)	Not done	SCID
Pt. 47	15 days/male	Younger sibling of pt. 31, pneumonia, recurrent diarrhea, failure to thrive	Blood culture: *Candida* sp.	0.094	N.A.	CD3- 42% (38) CD19- 40% (36) CD56- 16% (14)	*ADA*	SCID
Pt. 48	4 months/male	Younger sibling of pt. 27, recurrent pneumonia, diarrhea, failure to thrive, erythroderma, hepatosplenomegaly, eosinophilia (Omenn syndrome)	N.A.	1.896	N.A.	CD3- 74% (1,406) CD19- 0.4% (8) CD56- 22% (418) CD3+45RA+45RO-: 16% compared to 71% in control	*RAG2*	Omenn syndrome
Pt. 49	3 years/male	Recurrent sinopulmonary infections, diarrhea, failure to thrive, 1 episode of liver abscess, intra-cranial B cell lymphoma, defective T lymphocyte proliferation on stimulation with PHA.	N.A.	3.265	IgG- 4.02 g/L	CD3- 45.14% (1,467) CD3+CD4+- 6.9% (103) CD3+CD8+- 70.3% (1,033) CD19- 6.83% (222) CD56- 25.01% (816) CD3+45RA+45RO-: 71.06% compared to 64% in control CD3+CD4+45RA+45RO-: 3.6% compared to 72% in control CD3+CD8+45RA+45RO-: 75.3% compared to 68% in control	*CORO1A*	Atypical SCID
Pt. 50	6 months/male	Recurrent episodes of pneumonia, failure to thrive	N.A.	0.411	IgG <0.95 g/L IgA <0.17 g/L IgM <0.25 g/L	CD3- 20% (80) CD19- 73% (292) CD56- 1.4% (5-6)	*JAK3*	SCID
Pt. 51	10 months/male	Pneumonia, diarrhea, failure to thrive, meningoencephalitis	Endotracheal aspirate: *Klebsiella pneumoniae*	0.810	IgG <0.95 g/L IgA <0.17 g/L IgM <0.25 g/L	CD3- 4% (32) CD19- 95% (760) CD56- 1% (8)	*IL2RG*	SCID
Pt. 52	3 months/male	Recurrent pneumonia, diarrhea, failure to thrive	N.A.	0.199	N.A.	CD3- 0.82% (2) CD19- 1.17% (2-3) CD56- 88.9% (178)	*DCLRE1C*	SCID
Pt. 53	5 months/male	Pneumonia, failure to thrive, complicated otitis media with facial nerve palsy, transfusion-associated GVHD	N.A.	0.292	N.A.	CD3- 0.2% (0-1) CD19- 29% (87) CD56- 60% (180)	Not done	SCID
Pt. 54	3.5 years/male	Severe eczema since early infancy, pustules, otitis media, pneumonia, chest wall abscess, eosinophilia (incomplete Omenn)	Pus culture- *Staphylococcus aureus*	1.244	IgG- 1.64 g/L IgA- 1.56 g/L IgE- 4269 kU/L (upto 32) IgG1- 1.01 g/L IgG2- 0.95 g/L IgG3- 0.23 g/L IgG4- 0.71 g/L	CD3- 60% (744) CD3+CD4+- 17.3% (128) CD3+CD8+- 71.5% (529) CD19- 2.3% (28) CD56- 15% (186) CD3+45RA-45RO-: 36.6% compared to 65% in control CD3+45RA-45RO+: 67% compared to 31% in control CD3+HLA-DR+: 64.2% compared to 19.3% in control	No gene variants found	Omenn syndrome
Pt. 55	5 months/male	Recurrent pneumonia, diarrhea, failure to thrive, hyperferritinemia, hypofibrinogenemia, pancytopenia (HLH)	ET aspirate: *Klebsiella pneumoniae*, *Acinetobacter baumanii;* PCR positivity for H1N1	1.547	IgG- 2.32 g/L IgA <0.2 g/L IgM- 0.22 g/L	CD3- 1.74% (27) CD19- 91.6% (1,426) CD56- 5% (78)	IL2RG	SCID
Pt. 56	6 months/female	Pneumonia, failure to thrive, diarrhea, BCG site ulceration	N.A.	1.098	IgG- 0.54 g/L IgA <0.2 g/L IgM <0.17 g/L	CD3- 0% (0) CD19- 2% (22) CD56- 79% (869)	*DCLRE1C*	SCID
Pt. 57	7 months/male	Pneumonia, diarrhea, failure to thrive, hepatosplenomegaly, BCG site ulceration	N.A.	0.855	N.A.	CD3- 5.1% (43) CD19- 77.5% (667) CD56- 17% (146)	Not done	SCID
Pt. 58	11 months/male	Recurrent pneumonia, failure to thrive, hepatosplenomegaly, generalized adenopathy, BCG site ulceration, erythematous rash (incomplete Omenn), meningitis with hydrocephalus	Disseminated BCGosis, CMV DNA PCR positive, Endotracheal aspirate: *Klebsiella pneumoniae*	1.832	IgG- 7.74 g/L IgA- 0.36 g/L IgM- 2.42 g/L	CD3- 68% (1,244) CD3+CD4+- 7.8% (97) CD3+CD8+- 45.1% (558) CD19- 5.6% (102) CD56- 23.5% (430) CD3+CD4+45RA-45RO+: 90.4% compared to 30.2% in control CD3+HLA-DR+: 67.9% compared to 5.8% in control	*RAG1*	Omenn syndrome
Pt. 59	5 months/male	BCG site ulceration, persistent diarrhea, generalized papular rash	*M. bovis*	0.931	IgA<0.10 g/L	CD3- <1% CD19- 97% (902) CD56- <1%	*IL2RG*	SCID
Pt. 60	6 months/male	BCG site ulceration, oral thrush, septicemia	*Candida* sp.	2.129	N.A	CD3- 29% (617) CD19- 62% (1320) CD56- 8% (170)	No gene variants identified	SCID
Pt.61	5 months/female	BCG site ulceration, pneumonia, erythroderma, alopecia, CMV DNA PCR- positive	CMV, *M. bovis*	1.144	IgG-9.03 g/L IgA-0.17 g/L IgM-0.41 g/L	CD3-70.70% (806) CD19-0.14% (2) CD56-17.70% (202) CD3+45RA+-12.57% compared to 86% in control	*RAG2*	Omenn syndrome
Pt. 62	4 months/male	Severe pneumonia, CT chest: diffuse bilateral ground glass opacities with multifocal consolidation	Nil	0.507	IgG- <2.02 g/L IgA- <0.17 g/L IgM- <25 g/L	CD3- 57.23% (290) CD19-0.05% (1) CD56-35.08% (179)	*RAG1*	SCID
Pt. 63	5 months/male	Severe pneumonia, CT chest: bilateral small random nodules	Nil	1.236	IgG- <2.03 g/L IgA- <0.17 g/L IgM- <0.25 g/L	CD3- 0.28% (4) CD19-96.20% (1193) CD56-0.51% (6)	*IL2RG*	SCID
Pt. 64	5 months/female	Persistent pneumonia- pneumothorax, oral thrush	*Candida* sp.	0.180	IgG- <2.03 g/L IgA- <0.17 g/L IgM- 0.33 g/L	CD3-0.16% (1) CD19-0.16% (1) CD56-74.40% (134)	*DCLRE1C*	SCID
Pt. 65	1.5 months/female	Left ear complicated otitis media, pneumonia, diarrhea	*S. aureus*	2.443	IgG-8.04 g/L IgA-0.75 g/L IgM-1.38 g/L	CD3-22.87% (559) CD19-73.60% (1776) CD56-1.43% (34)	No gene variants identified	SCID
Pt. 66	7 months/male	BCG adenitis, encephalitis	*M. bovis*	0.506	IgG- <2.05 g/L IgA- <0.17 g/L IgM- <0.26 g/L	CD3-18.19% (93) CD19-0.08% (1) CD56-77.24% (394)	*DCLRE1C*	SCID
Pt. 67	8 months/female	Recurrent diarhea, failure to thrive, pneumonia, axillary adenopathy	Nil	6.864	IgG-<2.03 g/L IgM->4 g/L	CD3-69.75% (4785) CD3+CD4+ - 32% of CD3+ lymphocytes (1530) CD3+CD8+ - 62% of CD3+ lymphocytes (2967) CD3+45RA+ - 24.3% compared to 85% in healthy control CD19-8.95% (617) CD56-2.57% (178)	No gene variants identified	SCID
Pt. 68	10 months/male	recurrent gastroenteritis, pneumonia, DCT+ autoimmune hemolytic anemia	CMV	2.200	IgG-3.33 g/L IgA- <0.17 g/L IgM- 1.19 g/L	CD3-86.75% (1910) CD19-0.64% (13) CD56-6.50% (143) CD3+CD45RA+ -38.2% compared to 79% in control	*NHEJ1*	Atypical SCID
Pt. 69	5 months/male	persistent pneumonia, absent BCG scar	Nil	0.287	IgG-3.47 g/L IgA- 0.21 g/L IgM-0.76 g/L	CD3-51.62% (150) CD19-31.30% (91) CD56-9.46% (28)	No gene variants identified	SCID
Pt. 70	4 months/male	Recurrent pneumonia, diarrhoea, generalized erythematous macular rash, CMV retinitis, seizures, GVHD skin lesions	CMV	4.921	IgG- <1.99 g/L IgA- <0.36 g/L IgM- <0.25 g/L	CD3-0.54% (25) CD19-0.54% (25) CD56-91.74% (4512)	*DCLRE1C*	SCID
Pt. 71	6 months/male	Recurrent pneumonia, otitis media, ulceration at BCG site, hepatosplenomegaly	*Enterococcus* sp.	0.816	IgA-0. 56 g/L	CD3-1.39% (12) CD19-90.95% (746) CD56-5.4% (44)	*IL2RG*	SCID
Pt. 72	11 months/male	Skin pustule and abscess, generalized erythematous macular rash, oral thrush	Nil	1.118	IgG-<0.90 g/L IgA- <0.21 g/L	CD3-24.80% (278) CD19-6.20% (69) CD56-67.6% (757)	*NHEJ1*	SCID
Pt. 73	3.5 months/male	Recurrent pneumonia, diarrhoea, generalized erythematous macular rash	Nil	2.420	IgG- 1.62 g/L IgA- 0.09 g/L IgM- 0.48 g/L	CD3-5% (121) CD19- 47% (1137) CD56- 42% (1016)	No gene variants identified	SCID
Pt. 74	3 months/male	Recurrent pneumonia, diarrhoea	Nil	1.643	IgG- 3.09 g/L IgA- <0.07 g/L	CD3-87.90% (1442) CD19-1.7% (28) CD56-2.2% (33) CD3+45RA+ -1.6% compared to 78% in control	*ADA*	Atypical SCID
Pt. 75	3 months/male	Recurrent pneumonia, diarrhoea, failure to thrive	Nil	2.862	IgG- 2.14 g/L IgA- <0.20 g/L	CD3-35.90% (1026) CD19-3.11% (89) CD56- 38% (1087)	No gene variants identified	SCID
Pt. 76	12 months/female	Recurrent pneumonia, diarrhoea, oral thrush	Nil	4.300	IgG- 3.28 g/L IgA- 1.46 g/L IgM- 2.99 g/L	CD3-3.29% (142) CD19-79.37% (3414) CD56-9.63% (413)	No gene variants identified	SCID
Pt. 77	1.5 months/female	Recurrent pneumonia, otitis media, generalized erythematous macular rash	*Pichia fermentans; E. coli*	4.720	IgG- 4.27 g/L IgA- <0.16 g/L IgM- 0.35 g/L	CD3-49.03% (2303) CD19-1.27% (61) CD56-37.44% (1765) CD3+45RA+ - 1.46% compared to 73% in control	*RAG1*	Omenn syndrome
Pt. 78	6 months/male	Recurrent pneumonia, diarrhoea, generalized erythematous macular rash	Nil	1.808	IgG- <2.02 g/L IgA- 0.20 g/L IgM-1.71 g/L	CD3-95.65% (1732) CD19-1.78% (32) CD56-0.53% (9) CD3+45RA+ - 11% compared to 86% in control	*IL2RG*	Atypical SCID
Pt. 79	6.5 months/male	Recurrent pneumonia, diarrhoea	Nil	0.600	IgG- 3.65 g/L IgA- 0.38g/L IgM- 0.41 g/L	CD3-29.54% (177) CD19-41.13% (247) CD56-18.87% (114)	No gene variants identified	SCID
Pt. 80	15 months/male	Recurrent pneumonia, diarrhoea, oral thrush	*Klebsiella pneumoniae*, CMV	7.191	IgG-2.02 g/L IgA-0.18 g/L IgM-0.46 g/L	CD3-12.36% (892) CD19-0.74% (53) CD56-51.5% (3703) CD3+45RA+ - 14.29% (decreased)	No gene variants identified	SCID
Pt. 81	42 months/female	Recurrent pneumonia, oral thrush	Nil	1.615	IgG-21.77 g/L IgA-1.23 g/L IgM-1.81 g/L	CD3-37.40% (606) CD4- 5.7% CD8- 14.7% CD19-22.6% (366) CD56- 46% (743)	No gene variants identified	SCID
Pt. 82	3 months/male	Recurrent pneumonia, failure to thrive, oral thrush, one elder female sibling expired due to pneumonia in early infancy	Nil	2.492	IgG-2.72 g/L IgA-0.09 g/L IgM-0.73 g/L	CD3-30% (747) CD19-9.10% (227) CD56- 41% (1021)	No gene variants identified	SCID
Pt. 83	3 months/female	Recurrent pneumonia, diarrhoea, otitis media, oral thrush	Nil	2.608	IgG-<2.02 g/L IgA-<0.17 g/L IgM-0.90 g/L	CD3-32.68% (853) CD19-29.79% (783) CD56-33.41% (872) CD3+ 45RA+ -2.23% (decreased)	No gene variants identified	SCID
Pt. 84	4 months/male	Recurrent pneumonia, diarrhoea, otitis media, ulceration at BCG site	Nil	0.663	IgG-<2.02 g/L IgA-<0.17 g/L IgM-<0.25 g/L	CD3-87.66% (579) CD19-0.05% (1) CD56-10.22% (66)	*RAG1*	SCID
Pt. 85	4 months/male	Recurrent pneumonia, severe erythroderma, developmental delay	Nil	1.441	IgG-<2.02 g/L IgA-<0.17 g/L	CD3-0.16% (3) CD19-94.81% (1365) CD56-0.67% (10)	*IL2RG*	SCID
Pt. 86	15 months/male	Recurrent pneumonia, generalized eczematoid macular rash, developmental delay, myopathy	Nil	14.84	IgG-13.16 g/L IgA-1.70 g/L IgM<0.26 g/L IgE- 8423 U/L	CD3-92.20% (13,683) CD19-2.85% (416) CD56-3.21% (475) CD4+45RA+ - 12.17% compared to 56% in control CD8+45RA+ - 18.6% compared to 72% in control	*STIM1*	CID
Pt. 87	6 months/male	Recurrent pneumonia, extensive eczematoid rash	CMV	6.556	IgG-13.75 g/L IgA-0.42 g/L IgM-1.88 g/L IgE- 622 U/L	CD3-53.90% (3536) CD4- 4.9% (320) CD8- 30.2% (1968) CD19-25.9% (1706) CD56-5.6% (368)	No gene variants identified	Omenn syndrome
Pt. 88	2.5 months/female	Recurrent pneumonia, diarrhoea, BCG site abscess	Nil	0.055	IgG-2.33 g/L IgA<0.17 g/L IgM<0.25 g/L	CD3-95.58% (48) CD19-0.07% (1) CD56-0.79% (1)	*ADA*	SCID
Pt. 89	4 months/female	Recurrent diarrhoea, otitis media, generalized erythematous macular rash, ulceration at BCG site	*Enterococcus faecalis*	2.352	IgG<2.02 g/L IgM<0.21 g/L	CD3-68.95% (1621) CD19-0.05% (1) CD56-25.37% (597) CD3+ 45RA+ -6.79% compared to 70% in control HLA DR in CD3+ - 74.2% compared to 15% in control	*RAG1*	Omenn syndrome
Pt. 90	4.5 months/male	Recurrent pneumonia,	Nil	0.724	IgG-<0.95 g/L IgA<0.17 g/L	CD3-2.41% (17) CD19-0.45% (4) CD56-90.91% (655)	*DCLRE1C*	SCID
Pt. 91	4 months/male	Recurrent pneumonia, chorioretinitis, failure to thrive, 3 maternal uncles died at early infancy due to severe infections	Blood CMV PCR positive	1.760	IgG < 2.7 g/L IgA <0.4 g/L IgM- 1.07 g/L	CD3- 1% (18) CD19- 61% (1,098) CD56- 1% (18)	*IL2RG*	SCID
Pt. 92	4 months/male	Oral thrush, pneumonia, failure to thrive	N.A.	0.650	N.A.	CD3- 0.4% (2-3) CD19- 97% (631) CD56- 0.4% (2-3)	*IL2RG*	SCID
Pt. 93	6 months/female	Recurrent pneumonia, CMV chorioretinitis	Blood CMV PCR positive	2.200	IgG <2.7 g/L IgA <0.4 g/L IgM <0.25 g/L	CD3- 7.6% (167) CD19- 1% (22) CD56- 40% (880)	*RAG2*	SCID
Pt. 94	4.5 months/male	Persistent pneumonia, failure to thrive, elder sibling died at 6 months due to severe pneumonia	N.A.	1.750	IgG <1.37 g/L IgA <0.26 g/L IgM <0.16 g/L	CD3- 4% (70) CD19- 47% (823) CD56- 25% (438)	*IL7RA*	SCID
Pt. 95	6 months/male	Recurrent pneumonia, failure to thrive	N.A.	2.000	IgG <1.37 g/L IgA <0.26 g/L IgM- 0.53 g/L	CD3- 20% (400) CD19- 80% (1,600) CD56- 0.1% (2)	*IL2RG*	SCID
Pt. 96	3 months/female	Oral thrush, septicemia	N.A.	0.720	IgG- 0.8 g/L IgA <0.26 g/L IgM <0.18 g/L	CD3- 5% (36) CD19- 7% (50) CD56- 53% (382)	*RAG2*	SCID
Pt. 97	5 months/male	Recurrent episodes of pneumonia, diarrhea, failure to thrive, oral thrush, BCG site ulceration, elder female sibling expired at 6 months due to recurrent infections	N.A.	0.850	IgG <2.7 g/L IgA <0.4 g/L IgM <0.25 g/L	CD3- 5% (43) CD19- 2.3% (20) CD56- 46% (391)	Not done	SCID
Pt. 98	3 months/female	Recurrent pneumonia, failure to thrive	N.A.	0.700	IgG <2.7 g/L IgA <0.4 g/L IgM <0.25 g/L	CD3- 6.5% (46) CD19- 3.6% (25) CD56- 34% (238)	Not done	SCID
Pt. 99	3 months/female	Recurrent pneumonia, failure to thrive	N.A.	0.680	IgG- 0.65 g/L IgA <0.5g/L IgM <0.25 g/L	CD3- 2% (14) CD19- 4% (27) CD56- 53% (360)	*DCLRE1C*	SCID
Pt. 100	3.5 months/male	Persistent pneumonia, failure to thrive	N.A.	4.911	IgG- 0.82 g/L IgA <0.26 g/L IgM <0.18 g/L	CD3- 9.4% (461) CD19- 0.4% (20) CD56- 90% (4,410)	Not done	SCID
Pt. 101	6 months/female	Persistent pneumonia, failure to thrive	N.A.	5.756	IgG- 6.7 g/L IgA <0.26 g/L IgM- 0.78 g/L	CD3- 2% (116) CD19- 41% (2,362) CD56- 47% (2,707)	Not done	SCID
Pt. 102	7.5 months/male	Recurrent pneumonia, diarrhea, failure to thrive	N.A.	0.750	IgG- 0.76 g/L IgA- 0.08 g/L IgM- 0.08 g/L	CD3- 26% (195) CD19- 67% (503) CD56- 6% (45)	*JAK3*	SCID
Pt. 103	1 month/male	Erythroderma, loss of eyelashes, eosinophilia (incomplete Omenn), failure to thrive, two siblings (one male and one female) expired in early infancy due to erythroderma, generalized lymphadenopathy, and severe infections	N.A.	3.358	IgG- 5.86 g/L IgA <0.26 g/L IgM- 0.36 g/L IgE >2,500 U/L	CD3- 9% (302) CD19- 48% (1,613) CD56- 11% (370)	No variants identified	Omenn syndrome
Pt. 104	5 months/female	Recurrent pneumonia, otitis media, failure to thrive, oral thrush, pancytopenia, hepatosplenomegaly, seizures, encephalopathy (HLH)	N.A.	1.890	IgG <2.7 g/L IgA <0.4 g/L IgM <0.25 g/L	CD3- 32% (605) CD3+CD4+- 87% (528) CD3+CD8+- 13% (78) CD19- 65% (1,229) CD56- 3% (57)	*SP110*	SCID
Pt. 105	11 months/male	Chronic diarrhea, failure to thrive	Stool culture: *Acinetobacter* sp.	0.691	IgG- 3.0 g/L IgA- 0.52 g/L IgM- 0.39 g/L	CD3- 13.6% (94) CD19- 56% (386) CD56- 28% (193)	*RAG1*	SCID
Pt. 106	4.5 months/male	Oral thrush, recurrent pneumonia, diarrhea, failure to thrive, BCG site ulceration, 3 maternal uncles died at early infancy due to repeated infections	N.A.	0.900	IgG- 1.1 g/L IgA- 0.05g/L IgM- 0.07g/L	CD3- 5% (45) CD19- 91% (819) CD56- 2% (18)	*IL2RG*	SCID
Pt. 107	5 months/male	Recurrent pneumonia, oral thrush, failure to thrive, BCG site ulceration, encephalopathy	N.A.	1.120	IgG <1.4g/L IgA <0.17g/L IgM <0.19g/L	CD3- 6% (66) CD19- 92% (1,012) CD56- 1% (11)	*JAK3*	SCID
Pt. 108	8 months/male	Recurrent pneumonia, diarrhea, failure to thrive	N.A.	0.380	IgG <1.4g/L IgA <0.17g/L IgM <0.19g/L	CD3- 0% CD19- 94% (357) CD56- 4% (15)	*IL7R*	SCID
Pt. 109	4.5 months/male	Persistent pneumonia, failure to thrive	N.A.	2.092	IgG <1.4g/L IgA <0.17g/L IgM- 0.24g/L	CD3- 0.8% (17) CD19- 98% (2,048) CD56- 0%	Not done	SCID
Pt. 110	4.5 months/male	Persistent pneumonia, recurrent diarrhea, skin abscess, failure to thrive, situs inversus, one elder sibling died at early infancy due to pneumonia	N.A.	0.780	IgG- 1.92 g/L IgA- 0.04 g/L IgM- 0.02 g/L	CD3- 0.04% CD19- 0.29% CD56- 96%	Not done	SCID
Pt. 111	5.5 months/female	Recurrent pneumonia, failure to thrive, 3 elder male siblings died within first year of life due to severe infections	N.A.	1.425	IgG- 0.42 g/L IgA <0.03 g/L IgM- 0.34 g/L	CD3- 1.2% (17) CD19- 71% (1,012) CD56- 25% (356)	*CD3D*	SCID
Pt. 112	6 months/female	Recurrent pneumonia, failure to thrive	N.A.	0.336	IgG <1.36 g/L IgA <0.25 g/L IgM <0.18 g/L	CD3- 0.2% (0-1) CD19- 0% CD56- 99% (335)	Not done	SCID
Pt. 113	3.5 months/female	Recurrent pneumonia, failure to thrive, one elder female sibling died at 4 months due to a probable infection	N.A.	2.210	IgG <1.36 g/L IgA <0.25 g/L IgM <0.18 g/L	CD3- 0.8% (18) CD19- 97.4% (2,153) CD56- 1% (22)	No variants identified	SCID
Pt. 114	8 months/male	Recurrent pneumonia, BCG site ulceration, failure to thrive, one elder male sibling died at early infancy due to pneumonia	Disseminated BCGosis	1.650	IgG- 0.15 g/L IgA <0.24 g/L IgM- 0.2 g/L	CD3- 0% CD19- 61% (1,007) CD56- 38% (627)	*IL7R*	SCID
Pt. 115	7 months/female	Persistent pneumonia, failure to thrive, two elder siblings (one male, one female) died in early infancy due to severe infections, one had disseminated BCGosis	N.A.	0.870	IgG <2.0 g/L IgA <0.3 g/L IgM <0.2 g/L	CD3- 15% (131) CD19- 0% CD56- 40% (350)	*DCLRE1C*	SCID
Pt. 116	7 months/female	Persistent pneumonia, failure to thrive, autoimmune hemolytic anemia	Disseminated CMV, pulmonary aspergillosis	1.700	IgG- 3.2 g/L IgA- 0.38 g/L IgM- 0.4 g/L	CD3- 4% (68) CD19- 0% CD56- 72% (1,224)	*RAG2*	SCID
Pt. 117	8 months/male	Recurrent pneumonia, persistent diarrhoea, BCG site ulceration	Disseminated BCGosis	2.400	IgG- 2.9 g/L IgA- 0.32 g/L IgM- 0.24 g/L	CD3- 0% CD19- 70% (1,680) CD56- 24% (576)	*CD3E*	SCID
Pt. 118	5 months/male	Recurrent pneumonia, failure to thrive	*Pneumocystis jirovecii* from endotracheal aspirate	3.200	IgG- 3.64 g/L IgA- 0.42 g/L IgM- 0.38 g/L	CD3- 2% (64) CD19- 64% (2,048) CD56- 1% (32)	*IL2RG*	SCID
Pt. 119	3.5 months/male	Recurrent pneumonia, septicemia	None	0.064	IgG- 1.49 g/L IgA- <0.26 g/L IgM- <0.16 g/L	CD3- 63% (27) CD19- 2.4% (1) CD56- 2.4%	*ADA*	SCID
Pt. 120	1 month 8 days/female	Recurrent pneumonia, cupping of ribs with blunting of lower end of scapula in radiology	None	0.160	IgG- 3.54 g/L IgA- <0.05 g/L IgM- <0.03 g/L	CD3- 32% (51) CD19- 8.9% (14) CD56- 58% (93)	*ADA* (probable); Gene sequencing not done	SCID
Pt. 121	10 months/male	Recurrent pneumonia, persistent diarrhoea, oral candidiasis	Adenovirus	0.582	IgG- 3.54 g/L IgA- <0.05 g/L IgM- <0.03 g/L	CD3-11% (47.8) CD19-68.7% (298.7) CD56-18% (79.2)	*JAK3*	SCID
Pt. 122	7 months/female	Recurrent pneumonia, persistent diarrhoea, septicemia	Rhinovirus, Blood- *Candida* sp.	0.952	IgG- 16.55 g/L IgA- 0.29 g/L IgM- 1.12 g/L	CD3-4.7% (12) CD19-0% CD56-91% (231)	Not done	SCID
Pt. 123	6 months/male	Recurrent pneumonia, persistent diarrhoea	Nil	0.780	IgG- 3.06 g/L IgA- 0.26 g/L IgM- 0.30 g/L	CD3-85% (665) CD19-3% (26) CD56-11% (87)	*ADA*	SCID
Pt. 124	6 months/male	Recurrent pneumonia, persistent diarrhoea, cellulitis, hepatosplenomegaly, panniculitis	*M. bovis*	0.370	IgG- 0.19 g/L IgA- <0.01 g/L IgM- 0.16 g/L	CD3-4.94% (22) CD19-84% (404) CD56-0.09% (3)	Not done	SCID
Pt. 125	36 months/male	Recurrent pneumonia, persistent diarrhoea	Nil	0.480	IgG- 11.80 g/L	CD3-33.3% (156.5) CD19-33.4% (156.3) CD56- 28.3% (112)	Not done	SCID
Pt. 126	7 months/female	Recurrent pneumonia, persistent diarrhoea, septicemia	Blood- *Acinetobacter baumanni, Candida* sp.	1.090	IgG- 9.80 g/L IgA- 0.17 g/L IgM- 0.43 g/L	CD3-0.35% (4) CD19-82.7% (1048) CD56- 4.56% (58)	Not done	SCID
Pt. 127	11 months/male	Recurrent pneumonia, persistent diarrhoea, otitis media	Ear pus- *P. aeruginosa*	0.824	IgG- 12.40 g/L	CD3-3.0% (26) CD19-84% (682) CD56- 3.56% (48)	Not done	SCID
Pt. 128	4 months/female	Recurrent pneumonia, persistent diarrhoea, otitis media, cellulitis	BAL- Adenovirus	0.160	IgG- 7.30 g/L IgA- 0.34 g/L IgM- 0.92 g/L	CD3-0% CD19-30% (75) CD56- 42% (103)	Not done	SCID
Pt. 129	8 months/male	Recurrent pneumonia, persistent diarrhoea, otitis media, septicemia	Blood- *S. aureus, P. aeruginosa*	0.340	IgG- 3.30 g/L IgA- 0.24 g/L IgM- 0.17 g/L	CD3-2.0% (6) CD19-93% (310) CD56- 4% (18)	*JAK3*	SCID
Pt. 130	22 months/male	Recurrent pneumonia, persistent diarrhoea, septicemia	Blood- *Streptococcus pneumoniae*	0.357	IgG- 18.80 g/L IgA- 1.62 g/L IgM- 0.85 g/L	CD3-5% (18) CD19-27% (97) CD56- 72% (222)	Not done	SCID
Pt. 131	60 months/female	Recurrent pneumonia, persistent diarrhoea, septicemia, microcephaly	Nil	0.760	IgG- 13.80g/L IgA- 0.34 g/L IgM- 1.20 g/L	CD3-4.0% (24) CD19-92.0% (696) CD56- 3% (18)	Not done	SCID
Pt. 132	4 months/male	Recurrent pneumonia, persistent diarrhoea	Nil	1.378	IgG- 9.60 g/L IgA- 0.22 g/L IgM- 0.55 g/L	CD3-4.0% (44) CD19-91% (986) CD56- 5.3% (58)	Not done	SCID
Pt. 133	30 months/male	Recurrent pneumonia, persistent diarrhoea, otitis media, septicemia	CMV	0.357	IgG- 10.70 g/L IgA- 0.30 g/L IgM- 0.79 g/L	CD3-5.0% (17.5) CD19-12% (93) CD56- 34.6% (124)	Not done	SCID
Pt. 134	8 months/male	Recurrent pneumonia, otitis media, septicemia	Nil	3.485	IgG- 6.80 g/L IgA- 0.31 g/L IgM- 0.43 g/L	CD3-1.0% (6) CD19-82% (2830) CD56- 18% (654)	Not done	SCID
Pt. 135	5 months/male	Recurrent pneumonia, persistent diarrhoea, septicemia	*Candida* sp.	3.240	IgG- 2.80 g/L IgA- 0.18 g/L IgM- 0.26 g/L	CD3-12% (388) CD19-0% CD56- 86% (2786)	Not done	SCID
Pt. 136	6 month/male	Two elder male sibling death at early infancy	Nil	0.300	N.A.	CD3-0.7% (1) CD19-97.6% (290) CD56-0.4% (1)	*IL2RG*	SCID
Pt. 137	6 months/male	Recurrent pneumonia, septicemia, eczematoid rash	*Candida* sp.	2.436	IgG- 2.70 g/L IgA- 0.35 g/L IgM- 0.36 g/L IgE- 24,200 U/L	CD3-66% (1610) CD19-26% (634) CD56-8% (195) CD3+45RO+ - 97.5% (elevated)	*CD3D*	Omenn syndrome
Pt. 138	2 months/female	Recurrent pneumonia, cellulitis, OS, abscess	*Candida* sp.	32.600	IgG- <0.33 g/L IgA- <0.06 g/L IgM- <0.04 g/L	CD3-87% (28,362) CD19-0% CD56-7.6% (2478)	Not done	Omenn syndrome
Pt. 139	1 months/female	Cellulitis, rash	N.A	1.230	IgG- 9.40 g/L IgA- <0.25 g/L IgM- N.A	CD3-1% (12) CD19-N.A. CD56-N.A.	Not done	SCID
Pt. 140	36 months/male	Recurrent pneumonia, persistent diarrhoea	Clostridium difficle, CMV	3.024	IgG- 12.90 g/L IgA- 1.53 g/L IgM- 0.56 g/L	CD3-56% (1680) CD19-1.4% (42) CD56-26% (780)	*RAG1*	Atypical SCID
Pt. 141	6 months/female	Recurrent pneumonia, septicemia	*Candida* sp., *Staphylococcus* sp.	2.405	IgG- <0.75 g/L IgA- 0.24 g/L IgM- N.A	CD3-0.6% (14) CD19-62.4% (1504) CD56-22.9% (552)	Not done	SCID
Pt. 142	8 months/male	Recurrent pneumonia, persistent diarrhoea, septicemia	candida	2.075	IgG- 0.09 g/L IgA- <0.26 g/L IgM- <0.16 g/L	CD3-1% (21) CD19-93% (1934) CD56-0.2% (4)	Not done	SCID
Pt. 143	8 months/male	Recurrent pneumonia	Nil	2.650	IgG- 7.62 g/L IgA- 0.25 g/L IgM- 0.64 g/L	CD3-18.36% (488) CD19-5% (133) CD56- N.A.	Not done	SCID
Pt. 144	7 months/female	Recurrent pneumonia, persistent diarrhoea, BCG site ulceration	Nil	1.090	IgG- 0.10 g/L IgA- 0.02 g/L IgM- N.A	CD3- 1.1% (12) CD19- 0% CD56- 22% (231)	Not done	SCID
Pt. 145	5 months/male	Recurrent pneumonia,	Nil	0.060	IgG- 1.08 g/L IgA- 0.10 g/L IgM- 0.14 g/L	CD3-0.01% (1) CD19-NA (151) CD56- 62.33% (206)	Not done	SCID
Pt. 146	7 months/male	Recurrent pneumonia,	Nil	4.200	N.A	CD3-18.31% (838) CD19-51.69% (2682) CD56- 15.9% (826)	Not done	SCID
Pt. 147	9 months/female	Recurrent pneumonia, Septicemia, disseminated BCGosis	*E. coli*, *M. bovis*	0.994	IgG- 0.06 g/L IgA- 0.26 g/L IgM- 0.30 g/L	CD3-1.53% (10) CD19-84.69% (692) CD56- 2.76% (23)	Not done	SCID
Pt. 148	16 months/male	Recurrent pneumonia, Septicemia	*Candida* sp.	1.316	IgG- 12.20 g/L IgA- 1.08 g/L IgM- 6.54 g/L	CD3-0.54% (2) CD19-0.64% (3) CD56- 10.93% (46)	Not done	SCID
Pt. 149	5 months/male	Recurrent pneumonia, persistent diarrhea, BCG site ulceration	Nil	1.5	IgG- 0.02 g/L IgA- 0.37 g/L IgM- 0.21 g/L	CD3- 0.1% (2) CD19- 0% CD56- 95% (1425)	Not done	SCID
Pt. 150	7 months/male	Recurrent pneumonia,	BAL – *M.* tuberculosis, *Pseudomonas* sp.	3.000	IgG- <2.0 g/L IgA- 0.10 g/L IgM- 0.90 g/L	CD3-0.20% (6) CD19-70% (2100) CD56- 36% (1080)	*CD3E*	SCID
Pt. 151	6 months/male	Recurrent pneumonia, oral thrush	Enterococcal sepsis	0.6	IgG- 1.24 g/L IgA- <0.01 g/L IgM- <0.01 g/L	CD3- 47.2% (283) CD19- 0.1% (1) CD56- 46% (276)	*RAG1*	SCID
Pt. 152	5 months/male	Recurrent pneumonia, persistent diarrhoea	Nil	1.099	IgG- <0.75 g/L IgA- <0.10 g/L IgM- 0.35 g/L	CD3-0% CD19-93% (1015) CD56- 2% (23)	*IL2RG*	SCID
Pt. 153	2 months/male	Recurrent pneumonia, septicemia	*Candida* sp. (blood)	0.080	IgG- 1.90 g/L IgA- <0.05 g/L IgM- <0.05 g/L	CD3-10.4% (2.4) CD19-5.6 (1.29) CD56- 64% (14.84)	*ADA*	SCID
Pt. 154	3 months/male	Acute fever, cough	Nil	0.323	IgG- <1.46 g/L IgA- <0.24 g/L IgM- 0.97 g/L	CD3-26% (84) CD19-65% (202) CD56-40% (129)	*ADA*	SCID
Pt. 155	7 months/male	Persistent diarrhoea	Nil	2.538	IgG-1.59 g/L IgA- <0.24 g/L IgM- <0.17 g/L	CD3-0% (0) CD19-86% (2183) CD56%-11% (279)	*IL7R*	SCID
Pt. 156	21 months/male	Meningoencephalitis, right chorioretinitis, left vitreal hemorrhage	CMV	0.508	N.A.	CD3-5% (25) CD19-12% (61) CD56-62% (315)	*PNP*	SCID
Pt. 157	5 months/male	Pneumonia	*Citrobacter* sp.	1.520	IgG- <1.34 g/L IgA- <0.28 g/L IgM- 0.25 g/L	CD3-0% (0) CD19-0% (0) CD56-96% (146)	*RAG1*	SCID
Pt. 158	24 months/male	Recurrent pneumonia, diarrhoea, meningoencephalitis	*E. coli*	16.624	N.A.	CD3-85% (14130) CD4- 7% (1164) CD8- 70% (11637) CD19-10% (1662) CD56- 4% (665) HLA-DR expression on B cells- 0%	*RFXANK*	CID
Pt. 159	5 months/male	Pneumonia, diarrhoea, rash	*S. epidermidis*	0.320	N.A.	CD3-1% (1) CD19-32% (102) CD56-14% (45)	*ADA*	SCID
Pt. 160	5 months, female	Pneumonia	*P. jirovecii*, H1N1	1.967	N.A.	CD3-2% (39) CD19-0% (0) CD56-95% (1869)	Not done	SCID
Pt. 161	3 months, female	Pneumonia, diarrhoea	Nil	0.203	N.A.	CD3-0% (0) CD19-0% (0) CD56- 82% (166)	Not done	SCID
Pt. 162	12 months/male	Recurrent diarrhoea, left empyema	Nil	1.958	IgG- 15.7 g/L IgA- 3.94 g/L IgM- 2.13 g/L	CD3-43% (842) CD4- 2% (39) CD8- 30% (387) CD19-15% (294) CD56-38% (744)	Not done	SCID
Pt. 163	7 months, male	Pneumonia, global developmental delay	*M. tuberculosis*	0.979	NA	CD3-7% (69) CD19-86% (842) CD56-3% (29)	Not done	SCID
Pt. 164	2 months, male	Scaly erythrodermic rash (OS)	Nil	3.854	IgG-1.89 g/L IgA-0.28 g/L IgM-2.08 g/L	CD3-55% (2120) CD4- 33% (1272) CD8- 11% (424) CD4+ 45RA+ - 3% (decreased) CD19-18% (694) CD56-25% (964)	Not done	Omenn syndrome
Pt. 165	12 months, male	Abscesses in lung, liver, oral thrush	Nil	0.548	IgG-5.63 g/L IgA- <0.70 g/L IgM- <1.07 g/L	CD3-42% (230) CD19-18% (694) CD56-20% (110)	Not done	SCID
Pt. 166	2 months, male	Recurrent pneumonia, rash	*Acinetobacter* sp.	1.620	N.A.	CD3-68% (1102) CD4- 12% (194) CD8- 36% (583) CD4+ 45RA+ - 0% CD19-21% (340) CD56-6% (97)	Not done	Omenn syndrome
Pt. 167	10 months, male	Chronic fever, pneumonia, hepatomegaly, pancytopenia	Nil	0.954	IgG- <1.34 g/L IgA- <0.28 g/L IgM- <0.17 g/L	CD3-24% (229) CD19-63% (601) CD56-7% (67)	Not done	SCID
Pt.168	12 months, male	N.A.	N.A.	3.080	IgG- <0.29 g/L IgA- 0.64 g/L IgM- 0.34 g/L	CD3- 4.5% (138) CD19- 71.3% (2197) CD56- 1.9% (60)	*IL2RG*	SCID
Pt.169	60 months, male	NA	NA	0.870	IgG- <0.10 g/L IgA- <0.001 g/L IgM- <0.01 g/L	CD3- 0.5% (6) CD19- 89.7% (1076) CD56- 7.8% (94)	*IL2RG*	SCID
Pt.170	7 months, male	NA	NA	0.330	IgG- 0.06 g/L IgA- 0.001 g/L IgM- 0.002 g/L	CD3- 7% (23) CD19- 0.3% (1) CD56- 80.9% (267)	*RAG1*	SCID
Pt.171	48 months, male	NA	NA	1.160	IgG-0.59 g/L IgA-0.005 g/L IgM-0.007 g/L	CD3- 31.6% (367) CD19- 35.3% (410) CD56- 31.4% (364)	*RAG1*	SCID
Pt.172	12 months, female	NA	NA	7.220	IgG-2.36 g/L IgA-0.002 g/L IgM-0.01 g/L	CD3- 0.3% (22) CD19- 0.7% (54) CD56- 56% (4044)	*RAG2*	SCID
Pt.173	48 months, male	Otitis media, recurrent pneumonia since early infancy	NA	1.800	IgG-1.16 g/L IgA-0.008 g/L IgM-0.006 g/L	CD3- 9.4% (169) CD19- 58.3% (1049) CD56- 22.5% (406)	*DOCK2*	CID
Pt.174	4 months, female	Chronic diarrhoea, pneumonia, failure to thrive, absent thymus	*E. coli*, Cryptosporidium	1.63	IgG- 5.35 g/L IgA- 0.31 g/L IgM- 1.82 g/L	CD3- 89.7% (1462) CD19- 1.2% (19) CD56- 4% (65)	Not done	Possible SCID***
Pt.175	30 months, male	Recurrent pneumonia, diarrhoea, failure to thrive	Nil	0.35	IgG-7.02 g/L IgA-1.31 g/L IgM- 0.82 g/L	CD3- 56.3% (197) CD19- 0.8% (3) CD56- 39.4% (138)	Not done	SCID
Pt.176	9 months, male	Chronic diarrhoea, pneumonia, failure to thrive	*P. aeruginosa*, *Candida* sp.	0.60	IgG- 0.99 g/L IgA-0.7 g/L IgM-0.4 g/L	CD3- 35.8% (215) CD19- 6.7% (40) CD56- 47.8% (287)	Not done	SCID
Pt.177	14 months, female	Recurrent pneumonia, diarrhoea, failure to thrive	Nil	2.63	IgG-1.46 g/L IgA- <0.25 g/L IgM- <0.18 g/L	CD3- 74% (1947) CD4- 14% (368) CD8- 34% (895) CD19- 2% (53) CD56- 23% (605)	Not done	CID
Pt.178	3 months, male	Recurrent pneumonia, fungal skin infection, 2 early sibling death	*P. aeruginosa, Streptococcus* sp.	2.12	IgG-2.52 g/L IgA- <0.25 g/L IgM- 1.12 g/L	CD3- 57.3% (1215) CD4- 0.5% (11) CD8- 56.3% (1194) CD19- 38.2% (810) CD56- 1% (21)	Not done	SCID
Pt.179	7 months, male	Pneumonia, scaly erythrodermic rash	Nil	0.42	IgG- 0.18 g/L IgA-0.52 g/L IgM-0.42 g/L	CD3- 83.3% (350) CD19- 1% (4) CD56- 1% (4)	Not done	Omenn syndrome
Pt.180	14 months, male	Recurrent pneumonia, eczematoid rash, failure to thrive	Cryptosporidium	2.19	IgG- 2.16 g/L IgA-1.21 g/L IgM-1.22 g/L	CD3- 41% (897) CD4- 4% (88) CD8- 17% (372) CD19- 1% (22) CD56- 17% (372)	Not done	CID
Pt.181	6 months, male	Chronic diarrhoea, failure to thrive, septicemia	*E. coli, Candida* sp.	1.37	IgG-9.52 g/L IgA-1.79 g/L IgM-0.26 g/L	CD3- 87.1% (1194) CD4- 34% (466) CD8- 53.1% (727) CD19- 0.2% (3) CD56- 12% (165)	Not done	SCID
Pt.182	3 months, male	Meningitis, pneumonia, oral thrush, early sibling death	*P. aeruginosa*	3.55	N.A.	CD3- 35% (1244) CD4- 10% (355) CD8- 15% (533) CD19- 0.1% (4) CD56- 56% (1990)	Not done	SCID
Pt.183	16 months, male	Pneumonia, eczematoid rash, Varicella infection, early sibling death due to pneumonia	*Acinetobacter* sp., *Pseudomonas* sp.	2.55	N.A.	CD3- 46% (1174) CD4- 14% (357) CD8- 33% (842) CD19- 4% (102) CD56- 50% (1276)	Not done	CID
Pt.184	3 months, female	Pneumonia, abdominal distension, diarrhoea, failure to thrive	Nil	1.80	IgG-1.63 g/ IgA- <0.06 g/L IgM- <0.16 g/L	CD3- 35% (630) CD19- 0.8% (14) CD56- 63% (1134)	Not done	SCID
Pt.185	3 months, male	Pneumonia, failure to thrive	*M. tuberculosis*	0.50	IgG-9.03 g/L IgA- 0.39 g/L IgM-2.23 g/L	CD3- 45% (315) CD19- 50% (350) CD56- 1.4% (10)	Not done	SCID
Pt.186	9 months, male	Persistent diarrhoea, pneumonia, left forearm abscess	Nil	2.43	IgG-4.0 g/L IgA-0.74 g/L IgM- 1.1 g/L	CD3- 43.9% (1068) CD3+CD4+- 26% (631) CD3+CD8+- 14% (340) CD19- 54.9% (1335) CD56- 1% (24)	Not done	Possible SCID***
Pt.187	4 months, male	Developmental delay, pneumonia, diarrhoea, failure to thrive, 1 early sibling death	*E. coli*	1.50	IgG-2.95 g/L IgA-0.07 g/L IgM- 1.04 g/L	CD3- 44.9% (674) CD19- 44.9% (674) CD56- 10% (150)	Not done	Possible SCID***
Pt.188	3 months, female	Otitis media, oral thrush, failure to thrive	Nil	1.86	IgG-2.95 g/L IgA-0.07 g/L IgM- 1.04 g/L	CD3- 9% (167) CD19- 0.5% (9) CD56- 87.8% (1633)	Not done	SCID
Pt.189	96 months, female	Recurrent pneumonia, ear discharge, failure to thrive	Nil	1.21	IgG-2.14 g/L IgA- 7.05 g/L IgM-1.54 g/L	CD3- 39% (473) CD19- 16% (194) CD56- 41.2% (498)	Not done	CID
Pt.190	24 months, female	Ear discharge, diarrhoea, scaly rash (Omenn phenotype)	Nil	8.75	N.A.	CD3- 80% (7003) CD4- 5% (438) CD8- 30% (2626) CD19- 2% (175) CD56- 14% (1226)	Not done	CID
Pt.191	1 month, female	Septicemia, 3 early siblings died at early infancy	Nil	2.89	N.A.	CD3- 63.9% (1847) CD4- 55.9% (1616) CD8- 8% (231) CD19- 18% (520) CD56- 15% (433)	Not done	SCID
Pt.192	6 months, male	Multiple hypodense lesions in liver and spleen, necrotic retroperitoneal lymph nodes	Nil	0.01	N.A.	CD- 0 CD19- 0 CD56- 0	Not done	SCID
Pt.193	7 months, male	Recurrent pneumonia, diarrhoea, early sibling death due to disseminated BCGosis	Acid-fast bacilli, *Candida* sp. (BAL)	2.15	N.A.	CD3- 0% CD19- 98.9% (2128) CD56- 0.3% (6)	Not done	SCID
Pt.194	2.5 months, male	Diarrhoea, ear discharge, pneumonia, dermatitis, knee joint swelling, axilla abscess, 1 elder sibling expired due to SCID	Blood, pus: *S. aureus* (Methicillin sensitive)	2.85	N.A.	CD3- 57% (1624) CD4- 17% (484) CD8- 31% (883) CD19- 0.3% (9) CD56- 40% (1140)	Not done	SCID
Pt.195	NA, male	Pneumonia, otitis media, septicemia	*Pseudomonas* sp.	1.51	IgG-4.0 g/L IgA-0.52 g/L IgM-0.32 g/L	CD3- 0.3% (5) CD19- 0 CD56- 4% (62)	Not done	SCID
Pt.196	3 months, male	Pneumonia, oral thrush	Nil	0.01	N.A.	N.A.	*ADA*	SCID
Pt.197	3 months, male	Recurrent pneumonia, 1 early sibling death	Nil	2.86	N.A.	N.A.	*IL2RG*	SCID
Pt.198	2 months, female	Pneumonia, colitis	Nil	4.40	IgG-0.89 g/L IgA- <0.24 g/L IgM- <0.17 g/L	CD3- 0.5% (22) CD19- 87.3% (3839) CD56- 2% (88)	*JAK3*	SCID
Pt.199	1.5 months, female	Pneumonia, oral thrush, 2 elder female siblings died at early infancy	Nil	1.50	N.A.	CD3- 0 CD19- 63.1% (947) CD56- 34.7% (521)	Not done	SCID
Pt.200	8 months, male	Recurrent pneumonia, BCGosis	Nil	N.A.	N.A.	CD3- 0% CD19- 64% (443) CD56- 31% (214)	*IL7RA*	SCID
Pt.201	8 months, female	Recurrent pneumonia, oral thrush, BCGosis	Nil	0.55	IgG- <0.06 g/L IgA- <0.24 g/L IgM- <0.17 g/L	CD3- 0 CD19- 16.7% (92) CD56- 65.3% (359)	Not done	SCID
Pt.202	7 months, female	Recurrent pneumonia, diarrhoea	CMV viremia, *Candida* sp.	2.48	IgG-0.97 g/L IgA-1.82 g/L	CD3- 70.8% (1755) CD4- 2.6% (65) CD8- 59% (1463) CD19- 30.2% (748) CD56- 22.3% (553)	Not done	SCID
Pt.203	24 months, male	Recurrent pneumonia, otitis media	*S. aureus* (Methicillin resistant)	1.60	IgG-1.61 g/L IgA-0.29 g/L IgM-0.29 g/L	CD3- 22% (352) CD19- 58% (928) CD56- 13% (208)	Not done	SCID
Pt.204	24 months, female	Chronic diarrhoea, pneumonia	Corona virus 229E, Alpha hemolytic streptococci (blood), esophageal candidiasis	1.16	IgG-1.14 g/L IgA-0.15 g/L IgM-0.27 g/L	CD3- 23.4% (272) CD19- 9.1% (105) CD56- 42.3% (491)	*RAG1*	SCID
Pt.205	192 months, male	Recurrent pneumonia, varicella infection, madarosis, Hodgkin lymphoma	Epstein Barr viremia	N.A.	N.A.	N.A.	*RAG1*	Atypical SCID
Pt.206	5 months, male	Recurrent pneumonia, diarrhoea, elder male sibling died in early infancy, 4 maternal uncles expired < 6 months age	Adenovirus	N.A.	N.A.	NA; CD132 expression very low in monocytes (0.2%) compared to normal expression in controls	Not done	SCID
Pt.207	5 months, male	Pneumonia, diarrhoea, ear discharge, oral thrush, rash, early sibling death	VAPP in stool, Enterovirus, Klebsiella (BAL), CSF- Enterovirus, Mycoplasma	0.39	IgG- <1.46 g/L IgA- <0.28 g/L IgM- 0.17 g/L	CD3- 28% (109) CD19- 1% (4) CD56- 68% (265)	*RAG2*	SCID
Pt.208	20 days, male	Pneumonia, diarrhoea, rash, renal abscess	Corona OC43, Rhinovirus	0.25	N.A.	CD3- 19% (47) CD19- 0 CD56- 24.4% (61)	*ADA*	SCID
Pt.209	5 months, female	Chest wall abscess, recurrent pneumonia, oral thrush, diarrhoea	*P. jirovecii*, Rotavirus (stool), Mycoplasma (nasopharyngeal aspirate)	0.97	IgG- <1.46 g/L IgA- <0.17 g/L IgM- <0.28 g/L	CD3- 1.3% (13) CD19- 0 CD56- 60% (581)	*RAG2*	SCID
Pt.210	6 months, male	Recurrent pneumonia, diarrhoea, scalp abscess, 1 male sibling death	CMV, Rhinovirus, Enterovirus	N.A.	IgG-0.26 g/L IgA-0.02 g/L IgM- 1.70 g/L	N.A.	*CIITA*	CID
Pt.211	84 months, female	Recurrent diarrhoea, oral ulcer, pneumonia, colitis	Nil	0.84	IgG-4.97 g/L IgA- <0.67 g/L IgM-1.7 g/L	CD3- 77% (649) CD19- 15.5% (130) CD56- 3% (28) HLA-DR expression in B cells- 0%	*RFX5*	CID
Pt.212	18 months, male	Recurrent pneumonia, diarrhoea, failure to thrive	VDPV, *M. tuberculosis*, Cryptosporidium, *Enterobacter* sp. (blood)	3.75	IgG- <1.41 g/L IgA- <0.24 g/L IgM-0.20 g/L	CD3- 53.04% (1989) CD4- 22% (826) CD19- 4% (150) CD56- 42% (1576)	Not done	CID
Pt.213	132 months, female	Recurrent pneumonia, diarrhoea, oral thrush, otitis media, meningitis	*Hemophilus influenzae* (CSF)	2.94	IgG-0.22 g/L IgA- <0.24 g/L IgM-0.44 g/L	CD3- 34.7% (1022) CD4- 16.7% (490) CD8- 13.7% (405) CD19- 34% (1001) CD56- 2.2% (64)	Not done	CID
Pt.214	4 months, male	Failure to thrive, recurrent pneumonia, diarrhoea	Nil	1.22	NA	NA	*JAK3*	SCID
Pt.215	7 months, male	Otitis media, septicemia	Staphylococcus aureus	6.23	IgG- <0.3 g/L IgA- <0.05 g/L IgM- 0.11 g/L	NA	*IL2RG*	SCID
Pt.216	8 months, male	Pneumonia, diarrhoea, rash	Nil	5.02	IgG- <0.11 g/L IgA- <0.05 g/L IgM- <0.11 g/L	NA	*IL2RG*	SCID
Pt.217	1 month, male	Failure to thrive, persistent diarrhea, perianal rash	Nil	0.97	IgG- 0.42 g/L IgA- 0.06 g/L IgM- 0.59 g/L	CD3- 4% (39) CD19- 39% (378) CD56- 54% (524)	Not done	SCID
Pt.218	2 months, female	Recurrent episodes of pneumonia and diarrhoea, failure to thrive, doing well after HSCT	Nil	NA	NA	CD3- 3476 (Very low CD4 counts with CD4/CD8 reversal) CD19- 1765 CD56- 156	Probable MHC Class 2 defect	CID
Pt.219	1 month, female	Recurrent episodes of pneumonia and diarrhoea	Nil	NA	NA	NA	*IL7R*	SCID
Pt.220	1 month, male	Recurrent episodes of diarrhoea and failure to thrive	Nil	NA	NA	NA	*IL2RG*	SCID

ESID, European Society for Immunodeficiencies; CMV, Cytomegalovirus; BCG, Bacillus Calmette-Guerin; BAL, Bronchoalveolar lavage; CSF, Cerebrospinal fluid; OS, Omenn syndrome; PJP, Pneumocystis jirovecii pneumonia; EBV, Epstein-Barr virus; VDPV, Vaccine-derived polio virus; VZV, Varicella zoster virus; AIHA, Autoimmune hemolytic anemia; VAPP, Vaccine-associated paralytic polio; CID, Combined Immune Deficiency.

Clinical details of patients 221-277 are previously reported (7).

***Possible SCID is categorized if patients did not fulfil the complete ESID definition, however, the treating team had a high index of suspicion based on clinical and immunological features.

Clinical profile of all patients was obtained along with family history and other demographic details. Clinical features included number of infections, type of infections, site of infections, organism involved, age of presentation, age of onset, presence of skin rash, BCG ulceration, history of administration of vaccines and complications, if any. Basic hematological, biochemistry, and immunological investigations including immunoglobulin profile and lymphocyte subsets were also recorded.

Analysis of lymphocyte subsets by flow cytometry had been carried out in most patients. Methodology for laboratory assay of lymphocyte subsets, naïve, memory T cells, HLA-DR expression, CD132 expression, CD127 expression, and lymphocyte proliferation assays at Post Graduate Institute of Medical Education and Research (PGIMER), Chandigarh and National Institute of Immunohematology (NIIH), Mumbai have been previously described (11, 12). Other centers performed conventional lymphocyte subsets (CD3, CD19, CD4, CD8, CD56) by flow cytometry in private laboratories.

Adenosine deaminase (ADA) levels and percentage of deoxyadenosine nucleotides (%dAXP) from dried blood filter paper spot were assayed at Duke University, North Carolina for patients with ADA deficiency SCID who were diagnosed at PGIMER, Chandigarh.

### Molecular Assays

Before the facility for in-house next-generation sequencing was made available in 2018, centre at PGIMER, Chandigarh had established academic collaborations with centers at Hong Kong (The University of Hong Kong), Japan (Kazusa DNA Research Institute, Kisarazu, Chiba; National Defense Medical College, Saitama), and USA (Duke University, North Carolina) for molecular work-up of patients. The centre at Hong Kong provided final molecular diagnosis for 12 patients (Pt. 8-10, Pt. 14-19, Pt. 21, Pt. 50-51) (**Table 1**). Molecular diagnosis for 4 patients was established at Kazusa DNA Research Institute, Japan (Pt. 3–6). Thirty-four (34) patients (Pt. 59–90, pt. 119, pt. 127) with SCID were worked-up for molecular diagnosis using NGS at National Defense Medical College, Saitama and Tokyo Medical and Dental University, Tokyo, Japan (Kato T et al. manuscript in submission). Final molecular diagnosis of a patient with *ADA* defect (pt. 22) was also established at Duke University, North Carolina.

Sanger sequencing for *IL2RG* and *RAG1/2* genes were initiated at PGIMER, Chandigarh (North India) in 2016. Sanger sequencing for patients with SCID at NIIH, Mumbai (West India) was previously described by Aluri et al. (7). Methodology for NGS at Christian Medical College, Vellore (South India) was described previously (13).

#### Next-Generation Sequencing (NGS) at PGIMER, Chandigarh

Next-generation sequencing (Ion Torrent, Thermo Fisher Scientific India Pvt. Ltd.) for clinical care was started in July 2018 at the Advanced Pediatrics Centre, PGIMER, Chandigarh. A targeted PID gene panel comprising 44 genes was used that covered 6 genes for SCID—*ADA*, *RAG1*, *RAG2*, *IL2RG*, *IL7RA*, and *LIG4*. Preparation of DNA target amplification reaction using 2-primer pools, amplification of target, combination of target amplification reactions, ligating adaptors to the amplicons and their purification was carried out as per the manufacturer’s protocol using Ion AmpliSeq™ Library kit plus (Catalog numbers 4488990, A35121 A31133, A31136, A29751, 4479790). Amplified library was quantified using Qubit™ 2.0 fluorometer instrument. Dilution that results in a concentration of ~100pm was then determined. Template preparation on Ion One Touch™ Instrument, recovery, washing and enrichment of template-positive ISPs was done as per the manufacturer’s protocol using Ion 520™ and Ion 530™ Kit-OT2 (catalog number A27751). Ion S5™ sequencer instrument was then initialized. Annealing of primers to enriched ISPs and chip loading was carried out using Ion 520 and 530 Loading Reagents OT2 Kit. Sequencing run was initiated and Torrent Browser was used to review results. Raw data were analyzed on Ion Reporter software and on integrative genome viewer.

NGS using a targeted gene panel was also performed for some patients (n = 6) in private laboratories (Medgenome Labs Pvt. Ltd., India).

#### NGS at Other Centers

Other centers in India obtained molecular testing results from private laboratories (Medgenome Labs Pvt. Ltd., India; Strand Genomics Pvt. Ltd., India; Neuberg Anand Diagnostics Pvt. Ltd., India). Illumina platform was used for sequencing in private laboratories with coverage of >80X. Sanger sequencing was used to confirm variants obtained by NGS.

#### Multiplex Ligation Probe Amplification (MLPA) Assay for DCLERC1 Exon 1-3 Deletion at PGIMER, Chandigarh

SALSA MLPA probe-mix P368 DCLRE1C kit was used in this protocol. MLPA was performed according to the instructions provided by the manufacturer (MRC Holland). 50–100ng/µL of DNA was denatured in thermocycler and hybridized with 1.5 µL of probe-mix along with 1.5µL of MLPA buffer. Content was mixed and incubated for 1 min at 95°C followed by incubation at 60°C for 18 h. After hybridization, probes were ligated using a ligase mix at 54°C for 15 min. Ligase was inactivated at 98°C for 5 min. PCR was performed using PCR primers, polymerase, buffers and required amount of water. Following conditions were used for amplifications—95°C for 20 s, 65°C for 80 s, for 35 cycles, followed by a final extension for 20 min at 72°C. ABI 3100 Genetic Analyzer (Applied Biosystems, Foster City, CA, USA) was used for capillary electrophoresis. Later, 0.7µL of PCR reaction, 8.9µL of HI-DI formamide, and 0.4µL of DNA standard LIZ 600 provided by GeneScan were mixed and then denatured for 2 min at 95°C. The sample was then loaded and MLPA data were analyzed using a Coffalyser software.

## Results

**Figure 1:**
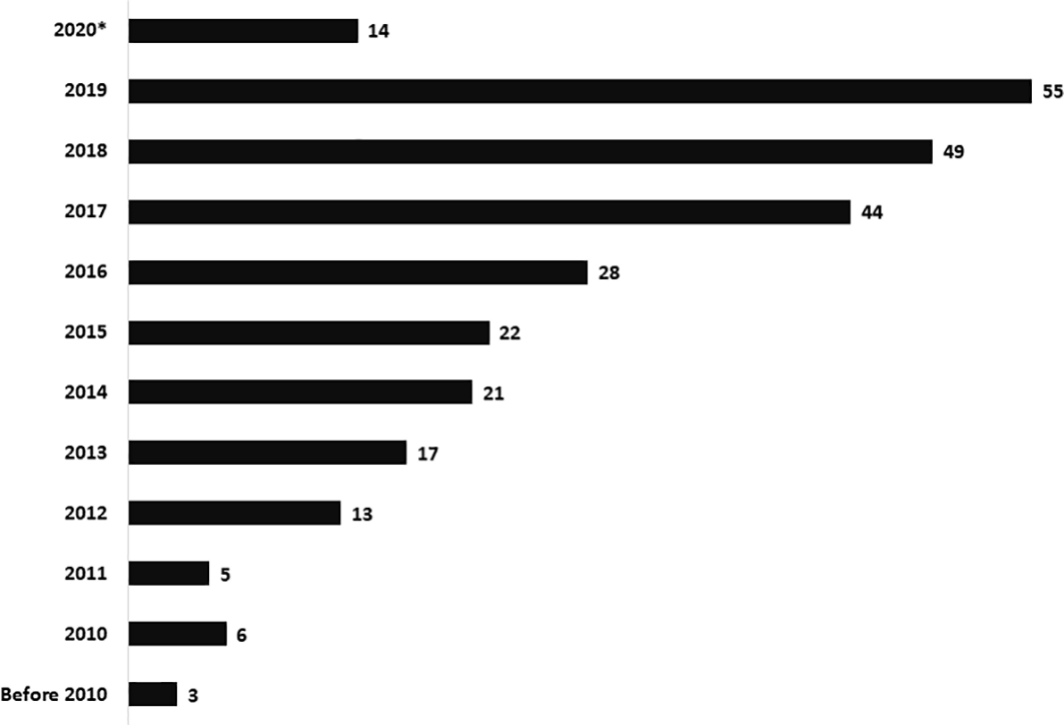
Bar graph depicting the rise in number of cases diagnosed over last 10 years.

Current study included data of patients diagnosed and managed at centers in Northern, Southern, and Western parts of India. Amongst the 277 patients, 254 were categorized as SCID (208 – SCID; 17 – atypical SCID; 26 – OS; 3 – possible SCID) and 23 as CID (**Table 1**). A steady increase in number of diagnosed cases was noted over last 10 years. The unit at PGIMER, Chandigarh (North India) diagnosed its first case of SCID in year 2001. Only 14 cases of SCID were identified until 2011 and an exponential rise in number of cases was noted after 2011 (**Figure 1**). Rise in number of cases over years paralleled the expansion of available manpower resources and laboratory facilities for pediatric immunology at Advanced Pediatrics Centre, PGIMER (North India). Ninety (90) children (Pt. 1-90) with SCID have been diagnosed at PGIMER, Chandigarh until date. Fifty-eight (58) and 27 cases of SCID were enrolled from Bai Jerbai Wadia Children’s Hospital, Mumbai (West India) and Aster CMI, Bengaluru (South India), respectively.

Male-female ratio was 196:81 (**Table 1**). Median [inter-quartile range (IQR)] age of onset of clinical symptoms and diagnosis was 2.5 months (1, 5) and 5 months (3.5, 8), respectively. Consanguinity was noted in 78 families (28.2%), and was noticeably more in Southern region (32.3%) of our country compared to Northern (22.4%). Family history of early childhood deaths was noted in 120 children (43.3%). Median (IQR) age at diagnosis in children who had a positive family history was 4.5 months (3, 6) compared to 6 months (4, 9) in children who did not have a family history, p<0.05 (Mann-Whitney U test).

**Figure 2:**
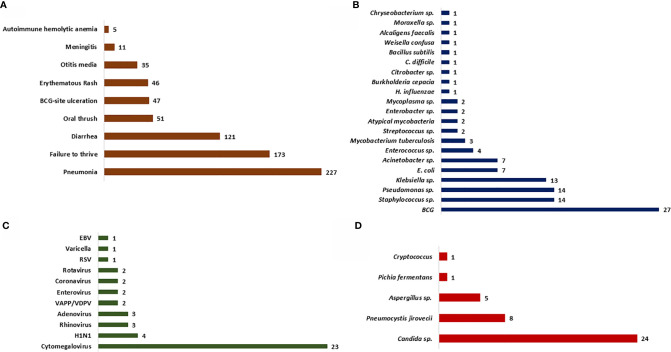
Bar graph depicting the clinical manifestations and microbiological profile. **(A)** Clinical manifestations noted at first clinical presentation; **(B–D)** Microbiological profile of the organisms isolated—bacteria **(B)**, fungi **(C)**, and viruses **(D)**.

**Figure 3:**
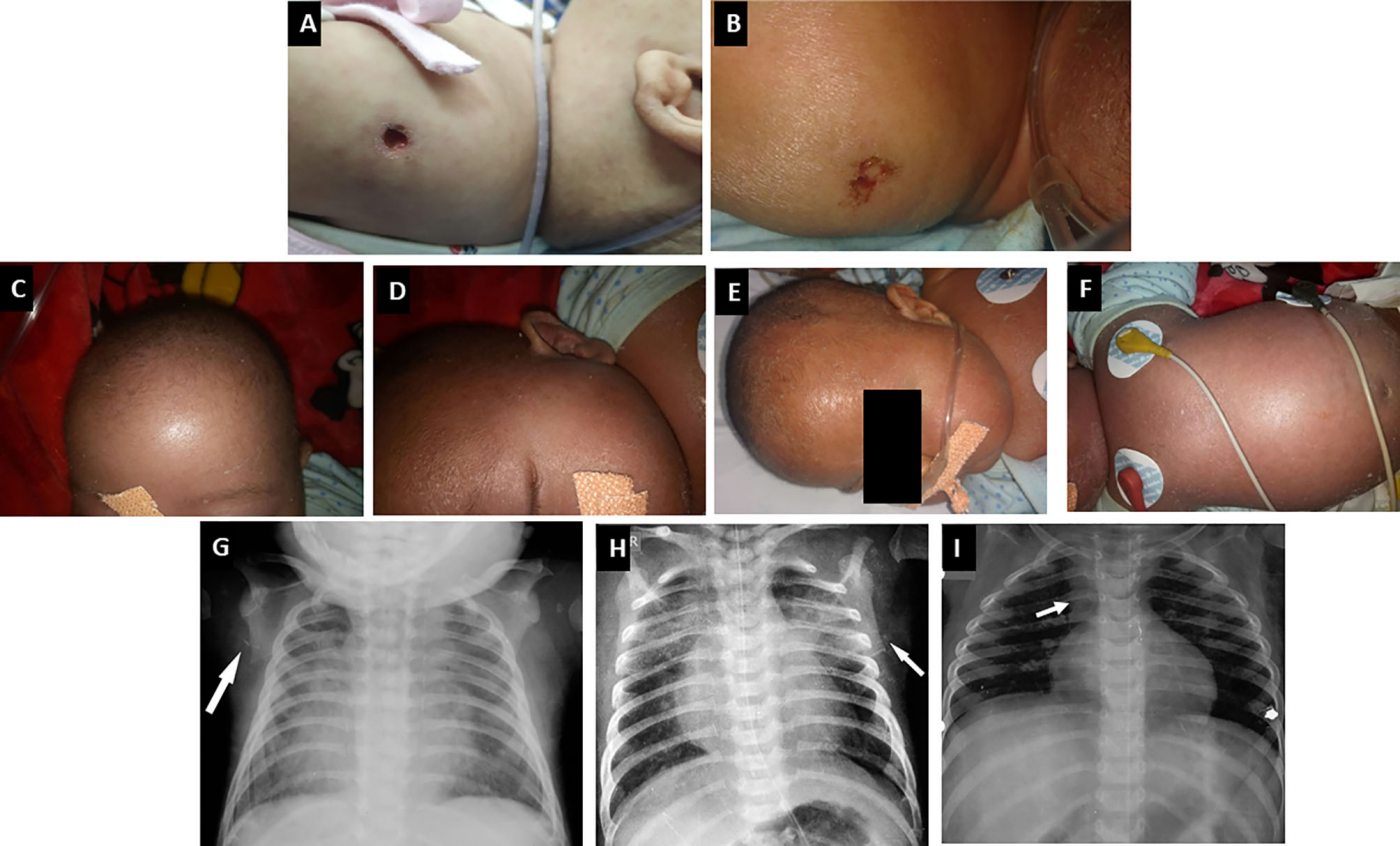
Clinical manifestations of children with SCID. **(A**, **B)** BCG site ulceration and pus discharge (Pt. 46 and 34); **(C–F)** Features of Omenn syndrome such as generalized erythema, scaling, loss of hair, and eyebrows (Pt. 34); **(G**, **H)** Chest radiograph of a child with ADA SCID showing radiological abnormalities—scapular spur and flattening of lower border of scapula (Pt. 39); **(I)** Chest radiograph of a child with *CORO1A* defect showing normal thymus shadow (Pt. 49).

Opportunistic infections were the presenting manifestation in most patients. These included pneumonia (82%), diarrhoea (43.7%), oral thrush (18.4%), BCG site ulceration (17%), otitis media (12.6%), and meningitis (4%) (**Figures 2**, **3**). Blood-culture proven septicemia was seen in 63 children (23%)—*Candida* sp. (16), *Staphylococcus* sp. (10), *Escherichia coli* (5), *Acinetobacter* sp. (5), *Pseudomonas aeruginosa* (8), *Klebsiella pneumoniae* (5), *Enterococcus* sp. (3), *Enterobacter* sp. (2), *Streptococcus* sp. (1), *Pichia fermentans* (1), *Burkholderia cepacia* (1), *Chryseobacterium* sp. (1), *Bacillus subtilis* (1), *Citrobacter* sp. (1), *Moraxella* sp. (1), *Alcaligens faecalis* (1), and *Weisella confusa* (1). Bacteria isolated from respiratory tract included *Mycobacterium bovis* (15), *Klebsiella pneumoniae* (5), *P. aeruginosa* (4), *M. tuberculosis* (3), atypical mycobacterium (1), *E. coli* (1), *Staphylococcus aureus* (1), and *Acinetobacter* sp. (1). Microbiology proven disseminated BCG infection was noted in 27 patients (9.7%). Apart from oral thrush and candidemia, other fungal infections noted were pneumonia due to *Pneumocystis jirovecii* (8), invasive aspergillosis (5), esophageal candidiasis (5), and pulmonary cryptcoccosis (1). Disseminated cytomegalovirus (CMV) infection was documented in 23 (8.3%) children and 6 amongst these had evidence of CMV retinitis. Intestinal lymphangiectasia due to CMV was noted on autopsy of a child with X-linked SCID (pt.8). Prolonged excretion of vaccine-derived poliovirus was documented in a child with leaky SCID at Mumbai (14, 15). Vaccine-associated paralytic poliovirus strain was also isolated in a child with *RAG1* defect at Mumbai. He had presented with persistent diarrhea, developmental delay, and hypotonia.

**Figure 4:**
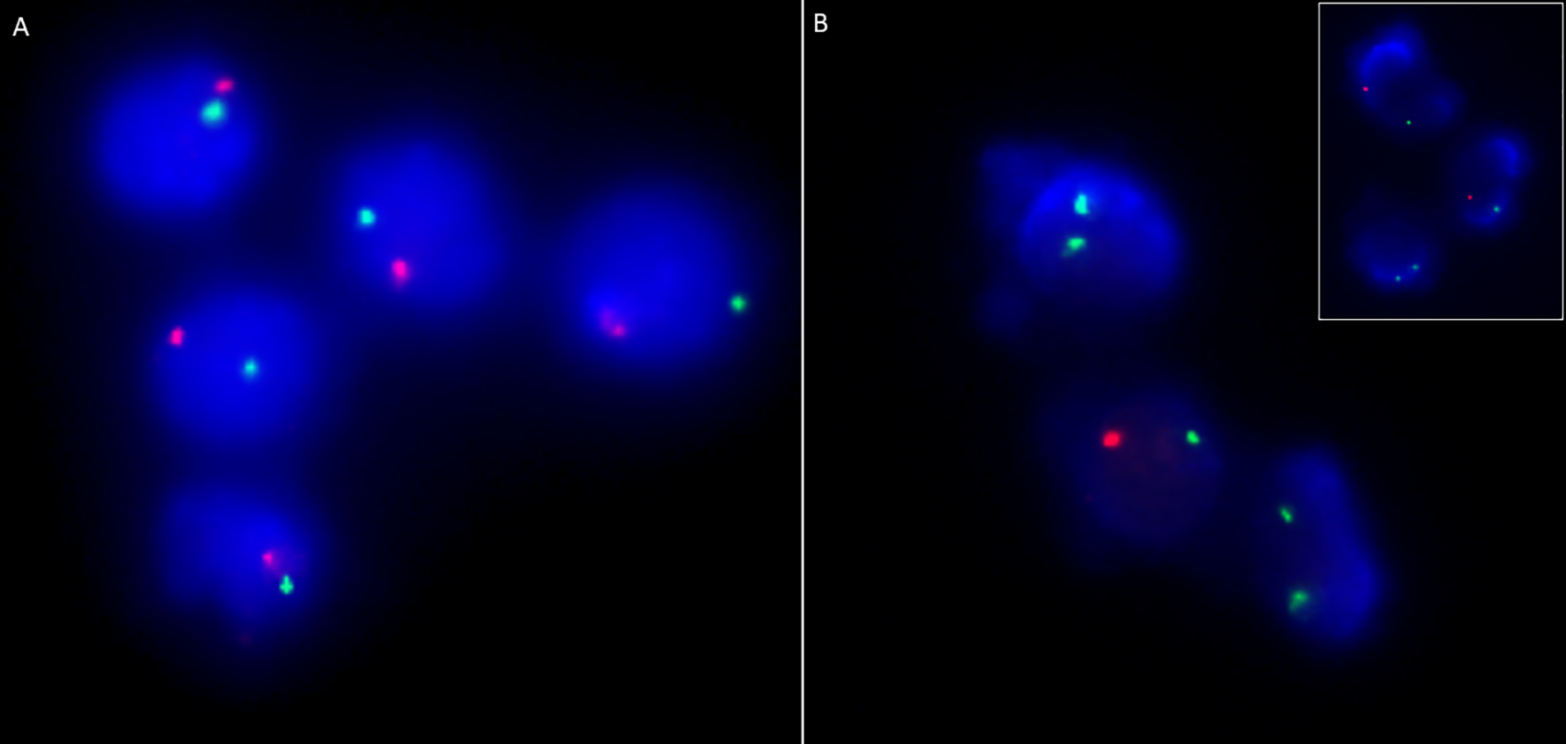
Chimerism analysis using dual colour FISH probes targeting centromeres of X (DXZ1; green) and Y (DYZ1, orange) chromosomes in a male child suspected with transplacental-acquired maternal T cell engraftment (Pt. 44). **(A)** Immunomagnetically sorted CD19 positive cells (B cells) showing XY pattern in all cells while; **(B)** Immunomagnetically sorted CD3 positive cells showing XX pattern in two out of three cells suggesting maternal T cell engraftment. Inset shows XX pattern in a lymphocyte and XY pattern in neutrophils.

Clinical features of OS were seen in 33 children (11.9%)—classical OS in 11 and incomplete OS in 22 (**Figure 3**). Molecular defects associated with OS include *RAG1* (7), *RAG2* (5), *ADA* (2), *NHEJ1* (1), *IL2RG* (1), *JAK3* (1), *STIM1* (1), *CD3D* (1), *DCLRE1C* (1), and *RFXANK* (1). Two children with *IL2RG* defect had features of engraftment of transplacental-acquired maternal T cells that mimicked clinical features of OS (**Figure 4**). Warm autoimmune hemolytic anemia (AIHA) requiring immunosuppressive medications was observed in 5 children. While anemia responded to intravenous (IV) methylprednisolone pulses in 2 patients (*RAG1* and *NHEJ1* defect each), pt.42 with *STK4* defect received IV rituximab (375 mg/m^2^ 2 doses) for control of AIHA and she did not have further relapse of AIHA for next 1.5 years. Transfusion-associated graft-vs-host reaction was documented in 4 patients (2 X-linked SCID; 2 AR-SCID); all had development of rash and transaminitis following transfusion of non-irradiated blood products. Four (4) children had features of hemophagocytic lymphohistiocytosis (HLH). Possible triggers for HLH included disseminated BCG (2) and H1N1 (1) infections. The child with *SP110* defect did not have any identifiable trigger for HLH (pt.104). Hodgkin lymphoma and intra-cranial B cell lymphoma were noted in children with *RAG1* and *CORO1A* defects, respectively.

Four of 18 children with *ADA* defect were noted to have radiographic abnormalities—scapular spurring and flattening of lower end of scapula (**Figure 3**). Glomerular involvement was seen in 4 children—3 children with OS and 1 with atypical/leaky SCID. Nephrotic range proteinuria was noted in 3 patients and one child (pt.13) had features of mesangial sclerosis on autopsy. Another child (pt. 12) with OS had features of focal segmental glomerulosclerosis on autopsy. One child (pt.10) with *IL7RA* defect had features of distal renal tubular acidosis and nephrocalcinosis. This patient had deletion of exons 2–5 of *CAPSL* along with exon 4–8 deletion of *IL7RA* in chromosome 5p13.2. A child with *PNP* defect (pt.14) had evidence of horse-shoe kidney at autopsy (16).

Median (IQR) absolute lymphocyte count (ALC) observed was 1.33 × 10^9^/L (0.6, 2.5). Normal ALC (≥ 3 × 10^9^/L) was observed in 51 children (18.4%)—of these 26 had OS, 2 had transplacental-acquired maternal T-cell engraftment, and 23 had leaky SCID/combined immunodeficiency. Eosinophilia was observed in 37 children, and 26 amongst these had features of OS. One child (Pt. 105) with *RAG1* defect had unexplained monocytosis (2.7-3.0 × 10^9^/L) that resolved after HSCT. Results of immunoglobulin profile was available for 198 children. Fifty-five (55) children had normal or elevated levels of IgM levels—30 in SCID (14.2%), 7 in atypical SCID (41.2%), 8 in OS (30.8%), and 10 in CID (43.5%). We observed elevated levels of IgE in 12 children—8 had OS, 1 had eczema and *STK4* defect, and 3 had unexplained eosinophilia.

Immunophenotyping by flow cytometry showed the following distribution: T-B-NK- (32), T-B+NK- (67), T-B+NK+ (33), T-B-NK+ (84). T+ SCID is observed in 20 children with OS—T+B-NK+ (17), T+B-NK- (2), T+B+NK+ (1) and 2 children with transplacental-acquired maternal T-cell engraftment—T+B+NK- (1), T+B-NK- (1). Genetic defects observed under each category are summarized (**Supplementary Table 2**). We observed decreased naïve (CD3+CD45RA+) and elevated memory (CD3+CD45RO+) CD3 lymphocytes in 24 children with OS. We noted elevated HLA-DR expression in CD3+ lymphocytes in 15 children with OS. CD132 expression by flow cytometry showed reduced expression in lymphocytes or monocytes in 8 children with suspected X-linked SCID (**Table 2**) (17–19). Levels of ADA and %dAXP were measured in 7 children with *ADA* SCID and 2 heterozygous carriers of *ADA* mutation (**Table 3**).

**Table 2:** CD132 expression by flow cytometry in children with X-linked SCID.

Patient	Molecular defect in *IL2RG*	Protein change	Type of mutation	Novel or previously reported	Clinical and Immunological phenotype	CD132 expression in case			CD132 expression in control		
						Lymphocyte	Monocyte	Neutrophils	Lymphocyte	Monocyte	Neutrophils
Pt. 25	c.202G>T (hemizygous); Mother - heterozygous carrier	p.E68X	Nonsense	Previously reported (17)	X-linked family history (5 maternal uncles died at early infancy), 1 elder male sibling (pt. 8) died at early infancy. T-B+NK- SCID	41.5%	94.1%	62.9%	50.9%	78.2%	39%
Pt. 35	c.170T>A (hemizygous); Mother -heterozygous carrier	p.L57H	Missense	Novel	Male child, T-B+NK- SCID, low CD132 expression	–	12.2%	–	–	87%	–
Pt. 40	–	–	–	–	X-linked family history (6 maternal uncles died at early infancy), 2 elder male siblings died at early infancy. T-B+NK- SCID; low CD132 expression	30.4%	60.3%	22.1%	84.1%	87.6%	30.5%
Pt. 43	c.455T>C (hemizygous); Mother -heterozygous carrier	p.V152A	Missense	Previously reported (18)	X-linked family history. Cousin brother of pt. 78. T-B+NK- SCID with low CD132 expression	–	15.8%	–	–	88.2%	–
Pt. 44	c.752C>G (hemizygous); Mother -heterozygous carrier	p.S251X	Nonsense	Novel	1 elder male sibling died at early infancy due to opportunistic infections. T+B+NK- SCID with low naïve CD3 cells and low CD132 expression	24.3%	25.8%	26.8%	48.3%	81.7%	77.5%
Pt. 46	c.596_598delinsTGGATTAT (hemizygous); Mother -heterozygous carrier	p.E199VfsX76	Frameshift	Novel	Male infant with T-B+NK- SCID with low CD132 expression	25.2%	98.2%	17.5%	83.5%	99.5%	66.3%
Pt. 59	c.8_9insA (hemizygous); Mother -heterozygous carrier	p.P4AfsX31	Frameshift	Novel (Kato et al., Manuscript in submission)	Male infant with T-B+NK- SCID with low CD132 expression; low naïve CD3 cells	51.1%	67.2%	66%	95.2%	98.9%	99.8%
Pt. 63	c.854G>A (hemizygous); Mother -heterozygous carrier	p.R285Q	Missense	Previously reported (19) (Kato et al., Manuscript in submission)	Male infant with T-B+NK- SCID with low CD132 expression	48.8%	50.4%	21.7%	84.1%	90.7%	85.1%
Pt. 78	c.455T>C (hemizygous); Mother -heterozygous carrier	p.V152A	Missense	Previously reported (18) (Kato et al., Manuscript in submission)	X-linked family history. Cousin brother of pt. 43. T-B+NK- SCID	67.8%	59.7%	24.8%	88.4%	89%	29.3%
Pt. 85	c.116-1G>T (hemizygous); Mother -heterozygous carrier	–	Splice-site	Novel (Kato et al., Manuscript in submission)	X-linked family history – 2 maternal uncles died at early infancy with severe infections. T-B+NK- SCID with low CD132 expression	53.7%	55.7%	55.1%	96.1%	92%	90.8%

**Table 3:** Erythrocyte ADA levels and % dAXP measured in dried blood spots.

Patient	Molecular defect in *ADA*	ADA levels (nmol/h/mg)	% dAXP	PNP levels (nmol/h/mg)
Normal levels		26.4 ± 10.0	<1.0	1354 ± 561
Pt. 22	c.301C>T c.461 G>T	0.1	63.9	1025
Mother of pt. 22	c.461 G>T (heterozygous carrier)	10.8	0	808
Father of pt. 22	c.301C>T (heterozygous carrier)	9.6	0	834
Pt. 30	c.646G>A	0	51.4	1264
Pt. 31	c.478+6T>A	0.3	6.8	1151
Pt. 36	c.407G>A	0	21.1	1532
Pt. 39	c.845G>T	0	54.2	1316
Pt. 74	c.466C>T	0	NA	NA
Pt. 88	c.845G>T	0	NA	929

ADA, adenosine deaminase; AXP (dAXP), total adenosine (deoxyadenosine) nucleotides; PNP, purine nucleoside phosphorylase; NA, Not available.

% dAXP= (dAXP/AXP+dAXP) x100.

**Figure 5:**
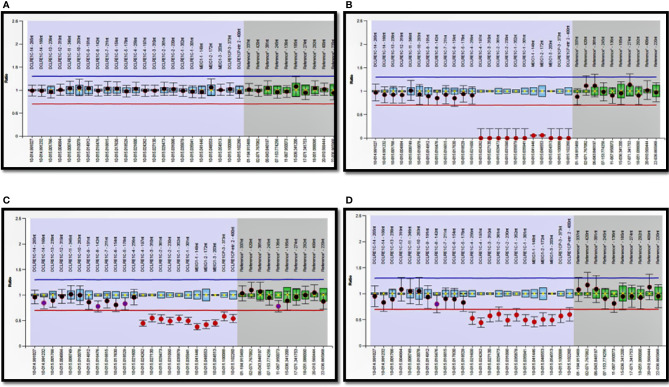
MLPA data was analyzed using Coffalyser software. **(A)** Healthy control sample having a Dosage Quotient (DQ) between 0.80 and 1.20; **(B)** A patient with T-B-NK+ SCID (pt. 66) showing a homozygous deletion and DQ=0; **(C**, **D)** Parents of index child showing a heterozygous deletion with DQ Score in range of 0.40 to 0.65.

Molecular diagnosis was obtained in 162 patients—*IL2RG* (36), *RAG1* (26), *ADA* (19), *RAG2* (17), *JAK3* (15), *DCLRE1C* (13), *IL7RA* (9), *PNP* (3), *RFXAP* (3), *CIITA* (2), *RFXANK* (2), *NHEJ1* (2), *CD3E* (2), *CD3D* (2), *RFX5* (2), *ZAP70* (2), *STK4* (1), *CORO1A* (1), *STIM1* (1), *PRKDC* (1), *AK2* (1), *DOCK2* (1), and *SP100* (1). Of the 176 molecular variants, 51 were identified to be novel in this study (**Table 4**, **Supplementary Table 3**) (7, 13, 17–49). A novel variant in *RAG1* (c.1758_1760delinsGAATC) was identified in 2 unrelated North Indian families. Deletion of exons 1-3 (8947bp) in *DCLRE1C* was observed in 11 children (9 from North and 2 from South India). MLPA confirmed EX1_EX3del in *DCLRE1C* in 7 children from North India (**Figure 5**). Targeted clinical exome sequencing by NGS did not identify pathogenic variants in 25 patients. Whole exome sequencing was performed in 5 children, and pathogenic variants were detected in 2 amongst these (pt. 50 and 51).

**Table 4:** Molecular defects in genes associated with SCID/CID in our cohort.

Pt No	Gene	Type of mutation	Exon	cDNA position	Protein change	Novel or previously reported	References
**1. SCID**							
Pt. 3	*IL2RG*	Hemizygous- missense	Exon 4	c.515T>G	p.L172R	Novel	Current study
Pt. 4	*IL2RG*	Hemizygous- nonsense	Exon 5	c.737G>A	p.W246X	Previously reported	(20)
Pt. 6	*IL2RG*	Hemizygous- missense	Exon 2	c.185G>A	p.C62Y	Novel	Current study
Pt. 8 and Pt. 25	*IL2RG*	Hemizygous- nonsense	Exon 2	c.202G>T	p.E68X	Previously reported	(17)
Pt. 35	*IL2RG*	Hemizygous- missense	Exon 2	c.170T>A	p.L57H	Novel	Current study
Pt. 43 and Pt. 78	*IL2RG*	Hemizygous- missense	Exon 4	c.455T>C	p.V152A	Previously reported	(18); (Kato T et al. Manuscript in submission)
Pt. 44	*IL2RG*	Hemizygous- nonsense	Exon 5	c.752C>G	p.S251X	Novel	Current Study
Pt. 46	*IL2RG*	Hemizygous- frameshift	Exon 5	c.596_598delinsTGGATTAT	p.E199VfsX76	Novel	Current study
Pt. 51	*IL2RG*	Hemizygous- nonsense	Exon 7	c.865C>T	p.R289X	Previously reported	(21)
Pt. 55	*IL2RG*	Hemizygous- nonsense	Exon 8	c.964C>T	p.Q322X	Previously reported	(22)
Pt. 59	*IL2RG*	Hemizygous- frameshift	Exon 1	c.8_9insA	p.P4AfsX31	Novel	(Kato T et al. Manuscript in submission)
Pt. 63	*IL2RG*	Hemizygous- missense	Exon 2	c.854G>A	p.R285Q	Previously reported	(19); (Kato T et al. Manuscript in submission)
Pt. 71	*IL2RG*	Hemizygous- splice site	Exon 4	c.594+5G>T		Previously reported	(22); (Kato T et al. Manuscript in submission)
Pt. 85	*IL2RG*	Hemizygous- splice site	Exon 2	c.116-1G>T		Novel	(Kato T et al. Manuscript in submission)
Pt. 91	*IL2RG*	Hemizygous- missense	Exon 2	c.854G>A	p.R285Q	Previously reported	(19)
Pt. 92	*IL2RG*	Hemizygous- nonsense	Exon 4	c.505C>T	p.Q169X	Novel	Current study
Pt. 95	*IL2RG*	Hemizygous- missense	Exon 5	c.677G>A	p.R226H	Previously reported	(23)
Pt. 106	*IL2RG*	Hemizygous- nonsense	Exon 1	c.67delC	p.L23X	Novel	Current study
Pt. 118	*IL2RG*	Hemizygous- splice-site	Intron 2	c.269+1G>T		Novel	Current study
Pt. 152	*IL2RG*	Hemizygous- missense	Exon 4	c.520T>A	p.W174R	Novel	Current study
Pt. 168	*IL2RG*	Hemizygous- missense	Exon 5	c.664C>T	p.R222C	Previously reported	(19)
Pt. 169	*IL2RG*	Hemizygous- missense	Exon 3	c.314A>G	p.Y105C	Previously reported	(24)
Pt. 197	*IL2RG*	Hemizygous- frameshift	Exon 3	c.359dupA	p.E121GfsX47	Novel	Current study
Pt. 216	*IL2RG*	Hemizygous- missense	Exon 5	c.670C>T	p.R224W	Previously reported	(22)
Pt. 220	*IL2RG*	Hemizygous- missense	Exon 5	c.664C>T	p.R222C	Previously reported	(19)
Pt. 18	*RAG1*	Homozygous- frameshift	Exon 2	c.1758_1760delinsGAATC	p.D587NfsX5	Novel	Current study
Pt. 19	*RAG1*	Homozygous- frameshift	Exon 2	c.908delC	p.P303LfsX42	Novel	Current study
Pt. 23	*RAG1*	Homozygous- frameshift	Exon 2	c.1758_1760delinsGAATC	p.D587NfsX5	Novel	Current study
Pt. 28	*RAG1*	Homozygous- missense	Exon 2	c.2147G>A	p.R716Q	Previously reported	(25)
Pt. 38	*RAG1*	Homozygous- frameshift	Exon 2	c.1178delG	p.G393AfsX10	Previously reported	(17)
Pt. 58	*RAG1*	Compound heterozygous- frameshift, missense	Exon 2 Exon 2	c.2849delT c.1421G>A	p.I950MfsX28 p.R474H	Previously reported Previously reported	(7)
Pt. 62	*RAG1*	Homozygous- missense	Exon 2	c.2210G>A	p.R737H	Previously reported	(26); (Kato T et al. Manuscript in submission)
Pt. 77	*RAG1*	Homozygous- missense	Exon 2	c.2923C>T	p.R975W	Previously reported	(27); (Kato T et al. Manuscript in submission)
Pt. 84	*RAG1*	Homozygous- missense	Exon 2	c.2923C>T	p.R975W	Previously reported	(27); (Kato T et al. Manuscript in submission)
Pt. 89	*RAG1*	Homozygous- missense	Exon 2	c.1211G>A	p.R404Q	Previously reported	(28); (Kato T et al. Manuscript in submission)
Pt. 105	*RAG1*	Homozygous- nonsense	Exon 2	c.310C>T	p.Q104X	Novel	Current study
Pt. 140	*RAG1*	Homozygous- missense	Exon 2	c.2333G>A	p.R778Q	Previously reported	(27)
Pt. 151	*RAG1*	Homozygous- missense	Exon 2	c.1331C>T	p.A444V	Previously reported	(29)
Pt. 157	*RAG1*	Homozygous- missense	Exon 2	c.1871G>A	p.R624H	Previously reported	(30)
Pt. 170	*RAG1*	Homozygous- missense	Exon 2	c.2326C>T	p.R776W	Previously reported	(31)
Pt. 171	*RAG1*	Homozygous- nonsense	Exon 2	c.424C>T	p.R142X	Previously reported	(32)
Pt. 204	*RAG1*	Compound heterozygous- missense, missense	Exon 2	c.1421G>A; c.1442G>A	p.R474H; p.C481Y	Previously reported; Novel	(29)
Pt. 205	*RAG1*	Compound heterozygous- missense, missense	Exon 2	c.323G>A; c.1228C>T	p.R108Q; p.R410W	Previously reported; Previously reported	(33, 34)
Pt. 5	*RAG2*	Homozygous- missense	Exon 2	c.1247G>T	p.W416L	Previously reported	(35)
Pt. 15 and Pt. 21	*RAG2*	Homozygous- nonsense	Exon 2	c.921G>A	p.W307X	Previously reported	(29)
Pt. 17	*RAG2*	Homozygous- missense	Exon 2	c.1247G>T	p.W416L	Previously reported	(35)
Pt. 27 and Pt. 48	*RAG2*	Homozygous- missense	Exon 2	c.1247G>T	p.W416L	Previously reported	(35)
Pt. 61	*RAG2*	Homozygous- missense	Exon 2	c.1247G>T	p.W416L	Previously reported	(35) (Kato T et al. Manuscript in submission)
Pt. 93	*RAG2*	Homozygous- missense	Exon 2	c.95G>A	p.G32E	Novel	Current study
Pt. 96	*RAG2*	Homozygous- missense	Exon 2	c.608G>A	p.G203E	Novel	Current study
Pt. 116	*RAG2*	Homozygous- missense	Exon 2	c.644C>T	p.T215I	Previously reported	(36)
Pt. 172	*RAG2*	Homozygous- frameshift	Exon 2	c.1056delA	p.D353MfsX91	Previously reported	(13)
Pt. 207	*RAG2*	Homozygous- missense	Exon 2	c.329T>C	p.M110T	Novel	Current study
Pt. 209	*RAG2*	Compound heterozygous- missense, frameshift	Exon 2	c.303T>G; c.171delG	p.N101K; p.K58SfsX73	Novel; Previously reported	Current study; (7)
Pt. 9	*DCLRE1C*	Homozygous- large deletion	Exon 1-3	EX1_EX3del		Previously reported	(37)
Pt. 20	*DCLRE1C*	Homozygous- large deletion	Exon 1-3	EX1_EX3del		Previously reported	(37)
Pt. 26	*DCLRE1C*	Homozygous- large deletion	Exon 1-3	EX1_EX3del		Previously reported	(37)
Pt. 52	*DCLRE1C*	Homozygous- large deletion	Exon 1-3	EX1_EX3del		Previously reported	(37)
Pt. 56	*DCLRE1C*	Homozygous- large deletion	Exon 1-3	EX1_EX3del		Previously reported	(37)
Pt. 64	*DCLRE1C*	Homozygous- large deletion	Exon 1-3	EX1_EX3del		Previously reported	(37); (Kato T et al. Manuscript in submission)
Pt. 66	*DCLRE1C*	Homozygous- large deletion	Exon 1-3	EX1_EX3del		Previously reported	(37); (Kato T et al. Manuscript in submission)
Pt.70	*DCLRE1C*	Homozygous- large deletion	Exon 1-3	EX1_EX3del		Previously reported	(37); (Kato T et al. Manuscript in submission)
Pt. 90	*DCLRE1C*	Homozygous- large deletion	Exon 1-3	EX1_EX3del		Previously reported	(37); (Kato T et al. Manuscript in submission)
Pt. 99	*DCLRE1C*	Homozygous- frameshift	Exon 10	c.874dupA	p.M292NfsX33	Novel	Current study
Pt. 115	*DCLRE1C*	Homozygous- large deletion	Exon 1-3	EX1_EX3del		Previously reported	(37)
Pt. 22	*ADA*	Compound heterozygous- missense, missense	Exon 4 Exon 5	c.301C>T c.461 G>T	p.R101W p.C154F	Previously reported; Novel	(38); Current study
Pt. 30 and Pt. 47	*ADA*	Homozygous- missense	Exon 7	c.646G>A	p.G216R	Previously reported	(39)
Pt. 31	*ADA*	Homozygous- splice-site	Intron 6	c.478+6T>A		Novel	Current study
Pt. 36	*ADA*	Homozygous- missense	Exon 5	c.407G>A	p.G136D	Novel	Current study
Pt. 39	*ADA*	Homozygous- missense	Exon 9	c.845G>T	p.R282L	Previously reported	(40)
Pt. 74	*ADA*	Homozygous- missense	Exon 5	c.466C>T	p.R156C	Previously reported	(41); (Kato T et al. Manuscript in submission)
Pt. 88	*ADA*	Homozygous- missense	Exon 9	c.845G>T	p.R282L	Previously reported	(40); (Kato T et al. Manuscript in submission)
Pt. 119	*ADA*	Homozygous- deletion	Exon 2	EX2_del		Novel	(Kato T et al. Manuscript in submission)
Pt. 123	*ADA*	Homozygous- missense	Exon 11	c.1028T>C	p.L343P	Previously reported	(42)
Pt. 153	*ADA*	Homozygous- splice-site	Intron 10	c.975+2T>G		Novel	Current study
Pt. 154	*ADA*	Compound heterozygous- nonsense, missense	Exon 6 Exon 8	c.523C>T; c.716G>A	p.Q175X; p.G239D	Previously reported; Previously reported	(7, 43)
Pt. 196	*ADA*	Compound heterozygous- nonsense, missense	Exon 6 Exon 8	c.523C>T; c.716G>A	p.Q175X; p.G239D	Previously reported; Previously reported	(7, 43)
Pt. 208	*ADA*	Homozygous- nonsense	Exon 6	c.523C>T	p.Q175X	Previously reported	(7)
Pt. 29	*JAK3*	Compound heterozygous- missense, missense	Exon 8 Exon 6	c.1048C>T; c.704T>C	p.R350W; p.M235T	Novel Novel	Current study Current study
Pt. 50	*JAK3*	Compound heterozygous- frameshift, missense	Exon 2 Exon 10	c.115delC c.T1289C	p.Q39SfsX108 p.I430T	Novel Novel	Current study Current study
Pt. 102	*JAK3*	Homozygous- nonsense	Exon 19	c.2605C>T	p.Q869X	Novel	Current study
Pt. 107	*JAK3*	Homozygous- frameshift	Exon 22	c.3049_3050delCT	p.L1017VfsX24	Novel	Current study
Pt. 121	*JAK3*	Homozygous- missense	Exon 11	c.1442A>G	p.E481G	Previously reported	(44)
Pt. 198	*JAK3*	Homozygous- missense	Exon 13	c.1765G>T	p.G589C	Novel	Current study
Pt. 10	*IL7RA*	Homozygous- large deletion	Exon 4-8	EX4_EX8del		Novel	Current study
Pt. 16	*IL7RA*	Homozygous- nonsense	Exon 5	c.616C>T	p.R206X	Previously reported	(45)
Pt. 94	*IL7RA*	Homozygous- frameshift	Exon 5	c.623delT	p.I208TfsX244	Novel	Current study
Pt. 108	*IL7RA*	Homozygous- splice-site	Intron 5	c.707-1G>T		Novel	Current study
Pt. 114	*IL7RA*	Homozygous- missense	Exon 4	c.509G>C	p.R170P	Novel	Current study
Pt. 155	*IL7RA*	Homozygous- large deletion	Exon 4-8	EX4_EX8del		Novel	Current study
Pt. 200	*IL7RA*	Homozygous- missense	Exon 3	c.324T>G	p.C108W	Novel	Current study
Pt. 219	*IL7RA*	Homozygous- nonsense	Exon 5	c.616C>T	p.R206X	Previously reported	(45)
Pt. 14	*PNP*	Homozygous- nonsense	Exon 3	c.244C>T	p.Q82X	Previously reported	(46)
Pt. 156	*PNP*	Homozygous- splice-site	Intron 3	c.286-18G>A		Previously reported	(47)
Pt. 111	*CD3D*	Homozygous- nonsense	Exon 2	c.158C>A	p.S53X	Novel	Current study
Pt. 137	*CD3D*	Homozygous- splice-site	Intron 2	(IVS2-2A>G)		Previously reported	(48)
Pt. 117	*CD3E*	Homozygous- nonsense	Exon 6	c.288T>A	p.Y96X	Novel	Current study
Pt. 150	*CD3E*	Homozygous- splice-site	Intron 6	c.352+1G>A		Novel	Current study
Pt. 68	*NHEJ1*	Homozygous- frameshift	Exon 5	c.544_545delGA	p.E182TfsX3	Novel	(Kato T et al. Manuscript in submission)
Pt. 72	*NHEJ1*	Homozygous- frameshift	Exon 3	c.221_222delGT	p.C74SfsX4	Novel	(Kato T et al. Manuscript in submission)
Pt. 49	*CORO1A*	Homozygous- splice-site	Intron 7	c.862-2A>G		Novel	Current study
**2. CID**							
Pt. 210	*CIITA*	Homozygous- nonsense	Exon 16	c.3122C>A	p.S1041X	Novel	Current study
Pt. 158	*RFXANK*	Homozygous- frameshift	Exon 6	c.430dupC	p.L144PfsX37	Novel	Current study
Pt. 211	*RFX5*	Homozygous- missense	Exon 7	c.446G>A	p.R149Q	Previously reported	(49)
Pt. 173	*DOCK2*	Homozygous- nonsense	Exon 34	c.3430C>T	p.R1144X	Previously reported	(13)
Pt. 41	*STK4*	Homozygous- nonsense	Exon 10	c.1165C>T	p.Q389X	Novel	Current study
Pt. 104	*SP110*	Homozygous- nonsense	Exon 8	c.855G>A	p.W285X	Novel	Current study
Pt. 86	*STIM1*	Homozygous- missense	Exon 10	c.1285C>T	p.R429C	Novel	(Kato T et al. Manuscript in submission)

Molecular analysis results of patients 221–277 are previously reported (7).

**Figure 6:**
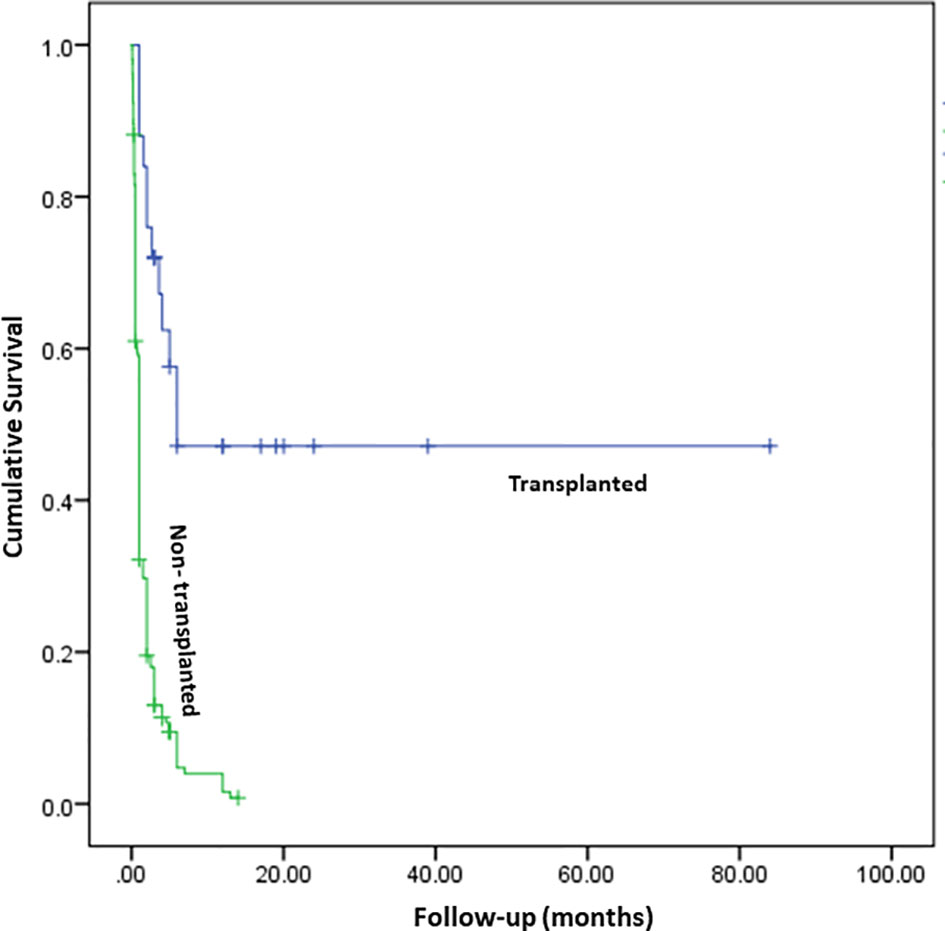
Survival curve comparing the outcome of children who underwent HSCT (n=23) and children who were not transplanted (n=213), p < 0.001 (Log Rank Mantel-Cox). Total person follow-up months—629.13 months.

Majority of patients (n=198) in this cohort succumbed to overwhelming infections as HSCT could not be carried out in them (**Figure 6**). Twenty-three patients (8.3%) underwent hematopoietic stem cell transplantation (HSCT) and 11 are doing well post-HSCT. The centre at South India (Apollo Children’s Hospitals, Chennai) has performed HSCT for 32 children with SCID until now and 17 are alive and doing well on follow-up. However, only 4 children are included in this analysis, as flow cytometry and mutation details were not available for other children. Another centre in South India (Aster CMI Hospitals, Bengaluru) has carried out HSCT for 9 children with SCID in the last 3 years (**Table 5**).

**Table 5:** Details of hematopoietic stem cell transplantation of 13 children with SCID.

****Patient	Type of SCID	Molecular defect	Centre	Age at transplantation	Donor characteristics	Outcome
Pt. 19	T-B-NK+	*RAG1*	PGIMER, Chandigarh	3.5 months	Father who is a complete HLA match with child	Developed BCG IRIS post-HSCT (D+90)- successfully treated with isoniazid, rifampicin, and ethambutol. He successfully engrafted and is currently doing well at 3^rd^ year follow-up.
Pt. 31	T-B-NK-	*ADA*	Diagnosed at PGIMER, Chandigarh; transplanted at Apollo Hospitals, Chennai	4 years	Fully matched unrelated donor	Engrafted and doing well at 1^st^ year follow-up.
Pt. 80	T-B-NK+	*-*	PGIMER, Chandigarh	18 months	Haploidentical donor	Developed graft failure. Underwent second transplant at 3 years (details not available).
Pt. 94	T-B+NK+	*IL7RA*	Aster CMI Hospitals, Bengaluru	5 months	Haploidentical (Mother)	Delayed graft failure (6 months post-HSCT). Underwent second HSCT with father being donor. Successfully engrafted and doing well at 20 months follow-up.
Pt. 103	T-B+NK+	N.A.	Aster CMI Hospitals, Bengaluru	1.5 months	Haploidentical (Mother)	Failed engraftment. Underwent second HSCT with father being donor- successfully engrafted, however, died after 1.5 months due to fulminant sepsis.
Pt. 105	T-B-NK+	*RAG1*	Aster CMI Hospitals, Bengaluru	11 months	Haploidentical (Mother)	Successfully engrafted. Doing well 1.5 years post-HSCT.
Pt. 106	T-B+NK-	*IL2RG*	Aster CMI Hospitals, Bengaluru	5 months	Haploidentical (Mother)	Successfully engrafted. Doing well 1.4 years post-HSCT.
Pt. 108	T-B+NK-	*IL7RA*	Aster CMI Hospitals, Bengaluru	8.5 months	Fully matched sibling	Died D+20 of HSCT- MDR Klebsiella sepsis.
Pt. 110	T-B-NK+	N.A.	Aster CMI Hospitals, Bengaluru	5 months	Haploidentical (Mother)	Successfully engrafted. Doing well 10 months post-HSCT.
Pt. 111	T-B+NK+	*CD3D*	Aster CMI Hospitals, Bengaluru	6 months	Haploidentical (Mother)	Expired D+9 due to pulmonary haemorrhage.
Pt. 114	T-B+NK+	*IL7R*	Aster CMI Hospitals, Bengaluru	9 months	Haploidentical (Father)	Developed severe gut GVHD and died D+60.
Pt. 115	T-B-NK+	*DCLRE1C*	Aster CMI Hospitals, Bengaluru	7.5 months	Haploidentical (Father)	Successfully engrafted. Doing well 2 months post-HSCT.
Pt. 152	T-B+NK-	*IL2RG*	Aditya Birla Memorial Hospital, Pune	5 months	Matched family donor	Doing well at 7^th^ year follow-up.

## Discussion

We describe the largest multi-centric cohort of patients with SCID from India. We included patients from 12 different tertiary care centers located in Northern, Southern, and Western parts of India. Patients from Eastern parts of India are usually referred to the centers located in other areas of India due to lack of availability of facilities for immunological investigations in that region. We witnessed an exponential rise in the number of cases with SCID after 2013 at multiple centers across India. We attribute this steady increase in cases to 2 factors—establishment of Indian Council of Medical Research Centers for Advanced Research in PIDs at PGIMER, Chandigarh (North India) and NIIH, Mumbai (West India) and expansion of laboratory facilities for pediatric immunology at other centers. The Pediatric Immunology and Bone Marrow Transplant Unit at Aster CMI Hospital, Bengaluru (South India) was established in 2017. Twenty-seven cases of SCID (Pt. 60–84) were diagnosed between 2017 and 2020, reflecting rise in awareness amongst referring pediatricians and better availability of diagnostic facilities at Bengaluru (South India).

Based on data from Sample Registration System of India, we estimated around 221 million live births from January 2011 to June 2020 (50). An estimated 257 patients with SCID have been diagnosed in this time period, which suggests a rough incidence of SCID at 0.12 per 100,000 live births. Though we have included data from most of the centers that care for patients with SCID in India, the estimated incidence from this study may not reflect true incidence of the country because of retrospective nature of the study and some patients diagnosed at other centers may have been missed. Nation-wide registry for SCID is needed for an accurate estimation of incidence. Nevertheless, if we extrapolate our current data on to the U.S. incidence figures of SCID (i.e. 1:58,000 live births), estimated number of children with SCID in India would be around 3,822 during the period 2011–June 2020 (1). Moreover, incidence of SCID in India is expected to be even higher than the U.S. considering high rates of consanguinity within the country. This suggests that though we have been increasingly diagnosing these children over the last few years, the diagnosis is still missed in almost 93% of these children. This is clearly unacceptable and mandates urgent intervention of health care professionals.

We observed a higher incidence of autosomal recessive forms of SCID (78.4%) compared to X-linked SCID. This is similar to reports from several other countries where consanguinity rates are high (**Table 6**) (7, 8, 51–59). Though consanguinity rate of 28.2% observed in our study is lower than that of Saudi Arabia and Iran, practice of endogamous and intra-community marriages is, perhaps, responsible for high proportion of autosomal recessive forms of SCID in India (2, 6). Median age at diagnosis of SCID in our study is 5 months. This is similar to reports from other countries such as China, Turkey, and U.S.A (**Table 6**). Children who had a family history of SCID had an earlier age of diagnosis (median:4.5 months) compared to children who did not have a suggestive family history (median:6 months). Our observation is similar to the report by Luk et al. that suggested the importance of family history for an early diagnosis of SCID (17).

**Table 6:** Comparison of our cohort with published multicentric studies on SCID from other countries in last 10 years.

Study (Year)	Place	No. of patients	Age of onset and diagnosis	Clinical manifestations	Molecular defects	Outcome
Yao et al. (51)	Shanghai, China	44; Male: female – 40:4	Mean age of onset – 3.56 ± 3.91 months Mean age at diagnosis – 7.1 ± 7.96 months	BCG-related complications noted in 14 children (31.8%). Three (3) had disseminated BCG infection. Two (2) had CMV infection	Defect in *IL2RG* noted in 25 children (56.8%).	Mortality seen in 37 children (84%). Six (6) children underwent HSCT and 1 of them had survived.
Pasic et al. (52)	Serbia and Montenegro	21	Median age of onset – 2 months	BCG-related complications in 41%. Pneumonia noted in 15 children (PJP- 5, CMV- 3, BCG-2, respiratory virus- 5). OS noted in 6 children.	17 had proven molecular defect (81%). *RAG1/2* commonest (12) followed by *IL2RG* (3), *JAK3* (2), *DCLRE1C* (1)	Mortality seen in 16 children (76.2%). Eight (8) children underwent HSCT and 5 of them survived.
Lee et al. (53)	South East Asia	42; Male: female – 30:12	Median age of onset and diagnosis – 2 and 4 months, respectively.	BCG-related complications in 10 children (23.8%) – 6 had localized reaction; 3 had regional adenitis; 3 had disseminated BCGosis. Oral thrush (12), CMV (2), and PJP (2) are other documented infections. OS noted in 4 children.	26 had proven molecular defect (61.9%). *IL2RG* commonest (19) followed by *IL7RA* (2), *JAK3* (2), *RAG1/2* (2), *DCLRE1C* (1)	12 children underwent HSCT and 8 of them survived.
Abolhassani et al. (54)	Iran	169; Male: female – 96:73	Mean age of onset and diagnosis – 4.2 and 8.6 months, respectively.	BCG-related complications noted in 23 (13.6%). Other infections noted are PJP (13), CMV (15), EBV (8), VDPV (6), Cryptococcus (6), and VZV (6). OS noted in 11 children.	37 had proven molecular defect (21.9%). *RAG1/2* commonest (19) followed by *IL2RG* (3), *JAK3* (3), *DCLRE1C* (3), *ADA* (2), *IL7RA* (2), *CD3E* (1), *CD3D* (1), *PRKDC* (1), *NHEJ1* (1), *PTPRC* (1)	NA
Rozmus et al. (55)	Canada	40	Mean age at diagnosis – 4.2 months.	Oral thrush (8), CMV (6), PJP (6), RSV (1), and adenovirus (1) are the infections noted.	20 had proven molecular defect (50%). *ADA* commonest (10) followed by *IL2RG* (4), *RAG1* (2), *ZAP70* (2), and MHC Class II defects (2).	Mortality observed in 12 children (30%). Fifteen (15) underwent HSCT and 10 of them survived.
Ikinciogullari et al. (8)	Turkey	234 (transplanted patients); Male: female – 145:89	Median age at diagnosis – 5 months.	Infections noted are oral thrush (51.5%), CMV (13.5%), bacterial infections (7.4%), BCG-related complications (2.2%), and respiratory viruses (4.4%)	42.3% had proven molecular defects – *RAG1/2* (15.4%), *JAK3* (6.8%), *IL2RG* (6%), *DCLRE1C* (5.6%)	Survival at 20 years is 65.7%
Mazzucchelli et al. (56)	Brazil	70; Male: female – 49:21	Mean age of onset and diagnosis – 3.3 and 6.7 months, respectively.	BCG-related complications seen in 39 children (55.7%) – disseminated form in 29 and localized in 10. Features of OS noted in 8 children.	NA	Mortality seen in 35 patients (50%). Thirty (30) underwent HSCT and 18 of them survived.
de Pagter et al. (57)	Netherlands	43	Median delay in diagnosis in typical and atypical SCID – 2 and 27 months, respectively.	Infections noted are bacterial sepsis (11), PJP (11), CMV (8), and BCGitis (6). AIHA seen in 5 children with atypical SCID.	*IL2RG* (21%), *RAG1* (21%), *RAG2* (5%), *ADA* (12%), *DCLRE1C* (7%), *PNP* (7%), and *IL7RA* (5%)	Mortality observed in 18 children (41.9%). Thirty-two (32) underwent HSCT and 24 of them survived. Two (2) underwent gene therapy and 1 survived.
Haddad et al. (58)	USA and Canada	662 (transplanted patients); Male: female – 471:191	Median age at diagnosis – 141.5 days (4.7 months)	NA	*IL2RG* (187), *RAG1/2* (52), *ADA* (45), *IL7RA* (40), *DCLRE1C* (28), *JAK3* (24), CD3 receptor defects (7), *PNP* (1), *AK2* (1), *CD45* (1)	Survival is better in children transplanted less than 3.5 months. Survival at 10 years is 71% and is higher with matched sibling donors compared to other donor types.
Micho et al. (59)	Greece	30; Male: female – 19:11	Median age at diagnosis – 6.2 months	NA	*DCLRE1C* (3), *IL2RG* (2), *JAK3* (2), *RAG1* (2), *ADA* (2), *PNP* (1)	Mortality is observed in 15 children (50%). Twenty-two (22) underwent HSCT and 14 of them are doing well.
Aluri et al. (7)	India	57; Male: female – 40:17	Median age of onset and diagnosis – 2 and 5.1 months, respectively	Infections observed include oral thrush (21%), BCG-related complications (12%), and PJP (1). OS noted in 4 children	49 children had proven molecular defects (86%). *RAG1/2* commonest (12), followed by *JAK3* (9), *IL2RG* (9), MHC Class II defects (6), *ADA* (5), *DCLRE1C* (2), *ZAP70* (2), *IL7RA* (1), *PRKDC* (1), *PNP* (1), and *AK2* (1)	Mortality observed in 47 children (82.5%). Four (4) underwent HSCT and none survived.
Present study (2020)	India	277 (23 CID, 254 SCID); Male: female – 196:81	Median age of onset and diagnosis – 2.5 and 5 months, respectively	BCG-related complications in 47 patients (17%) – localized form (20) and disseminated BCGosis (27). Other common infections include bacteria (72), CMV (23), Candida sp. (23), PJP (8), Aspergillus sp. (5), VAPP/VDPV (2). OS noted in 33 children. AIHA and lymphoreticular malignancy observed in 5 and 2 children, respectively.	162 patients had proven molecular defects (58.5%) - *RAG1/2* (43), *IL2RG* (36), *ADA* (19), *JAK3* (15), *DCLRE1C* (13), *IL7RA* (9), *PNP* (3), *CIITA* (2), *RFXAP* (3), *RFXANK* (2), *NHEJ1* (2), *CD3E* (2), *CD3D* (2), *RFX5* (2), *ZAP70* (2), *STK4* (1), *CORO1A* (1), *STIM1* (1), *PRKDC* (1), *AK2* (1), *DOCK2* (1), and *SP100* (1)	Mortality noted in 210 children (75.8%). Twenty-three (23) underwent HSCT and 11 of them are doing well.

BCG, Bacillus Calmette-Guerin; CMV, Cytomegalovirus; HSCT, Hematopoietic stem cell transplantation; OS, Omenn syndrome; PJP, Pneumocystis jirovecii pneumonia; EBV, Epstein-Barr virus; VDPV, Vaccine-derived polio virus; VZV, Varicella zoster virus; AIHA, Autoimmune hemolytic anemia; VAPP, Vaccine-associated paralytic polio; CID, Combined Immune Deficiency; SCID, Severe Combined Immune Deficiency.

Opportunistic infections in SCID are life-threatening and must be identified and treated adequately before HSCT. We documented a higher incidence of microbiologically-proven infections in our cohort compared to a previous report published from India (7). Amongst the bacterial infections, BCG was the commonest organism isolated. BCG-site ulceration has been noted in 47 children, however, disseminated BCGosis could be proven in 27 children only. BCG adenitis was noted in one child at D+90 post-HSCT as a part of immune reconstitution inflammatory syndrome. Lack of microbiological confirmation of BCG infection in many patients could have accounted for low rates of disseminated BCGosis in our cohort (**Table 6**) (51, 52). Infants with SCID who had received BCG vaccination and had not developed disseminated infection, are generally started on prophylactic medications—isoniazid and rifampicin at age-appropriate doses, that is generally continued until successful engraftment following HSCT.

Septicemia due to unusual organisms such as *W. confusa* and *A. faecalis* was also noted in our cohort. These are environmental bacteria and usually do not cause invasive infections in immunocompetent hosts. We also noted a high rate of disseminated CMV infection (8.3%) in our cohort. However, several amongst these were identified only on autopsy (60). This underscores the importance of vigilant screening and preventive measures for CMV infection in children with SCID. Cytomegalovirus infection, in our cohort, was possibly transfusion-acquired as most of the children received blood transfusions that are not always leuko-depleted and screened for active CMV infection. Though many patients had clinical features suggestive of *P. jirovecii* pneumonia (tachypnea, hypoxemia, interstitial pneumonia) and were treated for the same, microbiological or histopathological confirmation was possible in only 8 of them.

Thirty-three children had features of OS in our cohort. One child with OS (pt.54) was being treated as severe eczema for 3 years with multiple topical and systemic immunosuppressive agents, and diagnosis of SCID was made only after he developed severe infections. This highlights the importance of early identification of clinical phenotype of OS based on clinical features (generalized erythematous rash with scaling and partial loss of scalp hairs and eyebrows) and referral for appropriate immunological workup. Twenty-eight (28) children with OS had normal or high ALC and 2 children with transplacental-acquired maternal T-cell engraftment had elevated ALC. Laboratory assay of naïve T cells, memory T cells, and HLA-DR expression in T lymphocytes necessary for the diagnosis of OS are currently being performed only in two centers (PGIMER, Chandigarh and NIIH, Mumbai).

Twenty-three children in our cohort who did not have OS had normal ALC (>3 × 10^9^/L). However, lymphocyte subsets and naïve T cell estimation revealed diagnosis of SCID in them, thereby highlighting the importance of clinical suspicion and immunological investigations in infants with severe and life-threatening infections even if ALC is normal. Expansion of B cells or NK cells, engraftment of transplacental-acquired maternal T cells, or partial genetic defects allowing selective clone of T cell expansion could be the possible reasons for normal ALC in SCID. Aluri et al. have previously highlighted the importance of assessment of naïve T helper and cytotoxic T cells in children with severe infections and normal ALC to characterise MHC class II and *ZAP70* defects, respectively (7). A child with *IL7RA* defect in our cohort had a T-B+NK- phenotype, similar to the report by Aluri et al. (7). Also, two children with *IL2RG* defect had a T+B-NK- phenotype (1- OS, 1- transplacental-acquired maternal T cell engraftment). A possible explanation for low B cells is the depletion of B cells due to high inflammatory milieu secondary to OS and severe infections (61).

CD132 expression by flow cytometry is currently carried out at only two centers—PGIMER, Chandigarh (North India) and NIIH, Mumbai (West India). At PGIMER, Chandigarh, we found low CD132 expression in lymphocytes by flow cytometry as an inexpensive and rapid method of confirmation of diagnosis of X-linked SCID in 7 children. Two (2) children with X-linked SCID and previously reported variants in *IL2RG* (pt. 25 and pt. 78) had a normal expression of CD132 in lymphocytes (**Table 2**). We could not assay phosphorylated STAT5 in activated T-cells by flow cytometry to determine the functionality of IL2Rγ in many patients due to absent or very low amounts of T cells, however, naïve T cells by flow cytometry and TREC levels by RT-PCR have been assayed in some of them (**Table 2**). Only a handful centers in India (e.g. PGIMER, Chandigarh, North India, and NIIH, Mumbai, West India) have the wherewithal to perform functional studies. Both the centers have performed flow cytometry tests for samples received from other centers, however, timely transportation of viable blood samples from far off places, especially during hot summers remains a significant problem (11, 12). Lack of state-of-the-art facilities to do functional assays in all patients with SCID is one of the limitations of our study. Establishment of more clinical immunology laboratories, training of necessary manpower, and improvement in existing laboratory services are needed to overcome these barriers (11, 12).

Genetic confirmation of diagnosis of SCID is necessary for identification of pattern of inheritance and genetic counselling of affected families. Eighty-two (82) patients did not undergo a molecular analysis for confirmation of diagnosis due to lack of easy access to molecular diagnostics and financial difficulties. With the establishment of commercial NGS laboratories and reduction in costs involved for genetic sequencing over last few years, NGS-based diagnostics have become feasible in India (7, 13). In-house NGS facility for molecular diagnosis of PID is currently available only at PGIMER, Chandigarh (North India) and Christian Medical College, Vellore (South India). Most of the patients with SCID present in a critically-ill state and convincing families for genetic studies is often challenging due to significant financial and social constraints. It must be noted that expenses for molecular diagnosis are borne by the families in India most of the times as it is not covered by state or insurance schemes. Despite these challenges, we have been able to perform genetic studies in 195 patients. Academic collaborations with institutes at Hong Kong, Japan, and USA helped the centre at PGIMER, Chandigarh (North India) to get free molecular diagnosis for the families who cannot afford for costly molecular tests. We prefer to store blood samples in terminally-ill patients and later call the family for counselling to undergo genetic tests, as confirmation of molecular diagnosis has helped the families to undergo antenatal testing in subsequent pregnancies.

Defects in *RAG1/2* were found to be commonest in our cohort followed by *IL2RG*, *DCLRE1C*, and *ADA*. This is similar to the previous reports from Turkey, Iran, and Serbia (**Table 6**) (8, 52, 54). MHC Class II defect and defects in *STIM1*, *DOCK2*, *SP110*, *ZAP70*, and *STK4* genes are categorized as combined immunodeficiencies as per the 2019 International Union of Immunological Societies Expert Committee classification of human inborn errors of immunity (IEI) (5). However, we have included children with these defects in our cohort because they had severe infections from early infancy mimicking the clinical presentation of SCID (7).

Clinical phenotype of patients with *RAG1/2* defects in our cohort was very heterogenous. This included classical SCID, OS, atypical/leaky SCID phenotype, autoimmunity in form of AIHA, and development of hematological malignancy such as Hodgkin lymphoma. Wide spectrum of clinical manifestations could be due to difference in VDJ recombination activity or influence of other genetic or environmental factors (34, 62). Other reported clinical phenotypes in *RAG1/2* such as cutaneous granulomas, CVID-like phenotype or elevated γδ T cells were not seen in our cohort.

Low or undetectable ADA levels and elevated %dAXP levels were seen in 7 and 5 children with *ADA* defect, respectively. We noted that %dAXP levels in 2 children (pt. 31 and 36) were lower compared to other 3 children. While pt.31 had a milder clinical phenotype, pt.36 had features of OS. This suggests that low levels of accumulation of dAXP with residual ADA activity in lymphocytes may play a role in development of restricted T-cell clones that could be responsible for partial immunity and development of OS (63).

One child with *ADA* defect (pt. 36) had evidence of nephrotic syndrome along with OS. Renal abnormalities described with *ADA* defect (such as diffuse mesangial sclerosis) could result in nephrotic syndrome (64). However, renal involvement in OS manifesting as nephrotic syndrome has also been previously reported (65). We could not perform renal biopsy in this child due to severe ascites. Two other children with OS in our cohort also had renal involvement at autopsy—focal segmental glomerulosclerosis and mesangial sclerosis. Both of them also had severe infections—disseminated CMV in one and invasive aspergillosis in other. Whether the renal abnormalities are the result of genetic defect, inflammatory phenotype of OS, or severe infections is not clear and further research is needed in this regard.

Identification of radiosensitive forms of SCID is essential in B-NK+ SCID before HSCT as these children are prone to toxicity by chemotherapeutic drugs and radiation (66). Amongst the radiosensitive forms of SCID, molecular defects are predominantly noted in *DCLRE1C* in our cohort. Moreover, only mutation in *DCLRE1C* observed in North Indian children (n=9) was EX1_EX3 del. Initial MLPA screening for *DCLRE1C* exon 1-3 deletion before NGS in children with B-NK+ SCID was found to be more cost-effective than subjecting these children to NGS without a MLPA screen. The former approach is preferred at Chandigarh (North India) because of two reasons—NGS can miss large deletions and patients identified to have EX1_EX3 del in *DCLRE1C* by MLPA do not need to undergo NGS that is four to five times more expensive than MLPA in India. We also describe molecular defects in *STK4*, *CORO1A*, *CD3D*, *CD3E*, and *SP110* for the first time in India. Clinical phenotype of eczema, AIHA, and CD4 lymphopenia noted in *STK4* defect (pt. 41) has been previously described (67). Moshous et al. have described EBV-induced B cell lymphoma and naïve T-cell lymphopenia in patients with a hypomorphic missense variant in *CORO1A* (c.717G>A) (68). Our patient (pt. 49) with a novel splice-site defect in *CORO1A* (c.862-2A>G) had CD3 and CD4 lymphopenia, and developed an intracranial B cell lymphoma at 3.5 years of age.

A significant proportion of children (n=254) could not be subjected to HSCT due to medical and social reasons and succumbed to the illness. Presence of fulminant infections at time of diagnosis and lack of financial support dissuaded many families to undergo a costly procedure like HSCT. At present, facilities for pediatric HSCT for IEI are available at very few centers in India. Two centers in India have carried out most of the transplants for SCID – Apollo Children’s Hospitals, Chennai (South India) and Aster CMI Hospitals, Bengaluru (South India). Establishment of such dedicated pediatric HSCT units and development of manpower for HSCT services across the country are the need of the hour to ensure easy access to these services for affected patients. Provision of financial support from the government to affected families to undergo HSCT will also be required for successful outcomes. Studies from Western countries have shown that children with SCID transplanted below the age of 3.5 months of age had a significantly better outcome compared to children who underwent transplantation later (58). Though the age at diagnosis in our cohort is similar to countries where newborn screening has not been initiated, delayed referrals, presence of life-threatening infections at presentation, and lack of easy access to pediatric HSCT accounted for the unacceptable mortality rates in our cohort (**Figure 6**) (**Table 6**) (51, 52). We also realise that diagnosis of SCID is still being missed in most babies in India. Institution of universal newborn screening for SCID would provide more accurate estimates of incidence of SCID in our country and would also facilitate early diagnosis and treatment. However, financial implications and cost-effectiveness of implementing such a programme in a country as large, and as diverse, as India need to be worked out by health planners (69).

To conclude, we describe the largest multicentric cohort of SCID from India and document several novel mutations. Number of children with molecular diagnosis and those who have undergone HSCT has increased significantly in last decade. However, we are only too aware of our limitations. Improvement in awareness amongst physicians and pediatricians, expansion of diagnostic laboratories, institution of newborn screening, development of pediatric HSCT services, and financial support to the families to undergo HSCT are essentially needed for a better diagnosis and outcome of affected patients in the country.

## Data Availability Statement

The datasets presented in this study can be found in online repositories. The names of the repository/repositories and accession number(s) can be found in the article/ **Supplementary Material**.

## Ethics Statement

Ethical review and approval was not required for the study on human participants in accordance with the local legislation and institutional requirements. Written informed consent to participate in this study was provided by the participants’ legal guardian/next of kin. Written informed consent was obtained from the minor(s)’ legal guardian/next of kin for the publication of any potentially identifiable images or data included in this article.

## Author Contributions

PV, AS, AGum, JN, AJ, DS, AGup, AlK, MD, PT, VG, AP, SagB, SR, RC, MeS, DM, SarB, ArR, AA, FN, BJ, AM, HL, RU, RR, SanB, and SuS—Clinical management of patients; provided necessary clinical details for compilation. AmR, RK, MaS, AnK, BS, RM, KaS, AD, NJ, PK, MM, AV, KoS, SrS, YO, TK, KI, KC, DL, OO, SN, MH, and Y-LL—Laboratory work-up of patients; provided necessary laboratory results for compilation. KG—Provided necessary histopathology details. PV, RK, AS, AGum, MaS, AnK, and JN—Compiled the data and framed the initial draft and editing of manuscript. PV, RK—Literature search. PV, AmR, and SuS—Editing of manuscript at all stages of preparation and final approval. All authors contributed to the article and approved the submitted version.

## Conflict of Interest

The authors declare that the research was conducted in the absence of any commercial or financial relationships that could be construed as a potential conflict of interest.
