# Supplementary material for: Clinical, Immunological, and Molecular Features of Severe Combined Immune Deficiency: A Multi-Institutional Experience From India
Source: Front Immunol. 2021 Feb 8;11:619146. doi: 10.3389/fimmu.2020.619146 (PMC7897653; doi:10.3389/fimmu.2020.619146)
Supplement: Supplementary file 1 [file Table_1.docx]

**Supplementary table 1. Number of patients with SCID enrolled from each centres**

| **Centre** | **No. of patients** |
| --- | --- |
| Post Graduate Institute of Medical Education and Research, Chandigarh | 90 (Pt. 1-90) |
| Bai Jerbai Wadia Hospital for Children, Mumbai | 58* (Pt. 174-213) |
| ICMR-National Institute of Immunohaematology, Mumbai | 52** (Pt. 154-167) |
| Aster CMI hospital, Bengaluru | 27 (Pt. 91-115, pt. 119-120) |
| Kanchi Kamakoti Child Trust Hospitals for Children, Chennai | 16 (Pt. 121-135, pt. 149) |
| Institute of Child Health, Madras Medical College, Chennai | 8 (Pt. 143-148, pt. 150-151) |
| Sanjay Gandhi Postgraduate Institute of Medical Sciences, Lucknow | 8*** (Pt. 136-142) |
| Christian Medical College, Vellore | 6 (Pt. 168-173) |
| Zydus Hospitals, Ahmedabad | 3 (Pt. 116-118) |
| Kasturba Medical College, Mangalore | 4 (Pt. 214-217) |
| Apollo Children’s Hospital, Chennai | 3 (Pt. 218-220) |
| Aditya Birla Memorial Hospital, Pune | 2 (Pt. 152-153) |

*18 patients are already reported (Aluri et al) (7)

**38 patients are already reported (Aluri et al) (7)

***1 patient is already reported (Aluri et al) (7)
