# Supplementary material for: Clinical, Immunological, and Molecular Features of Severe Combined Immune Deficiency: A Multi-Institutional Experience From India
Source: Front Immunol. 2021 Feb 8;11:619146. doi: 10.3389/fimmu.2020.619146 (PMC7897653; doi:10.3389/fimmu.2020.619146)
Supplement: Supplementary file 2 [file Table_2.docx]

**Supplementary table 2. Flow cytometry-based classification of SCID and associated genetic defects**

| **Flow cytometry category (n)** | **Genetic defects (n)** |
| --- | --- |
| T-B+NK- (67) | *IL2RG* (26)  *JAK3* (12)  *SP110* (1)  *IL7R* (2) |
| T-B+NK+ (33) | *IL7R* (5)  *CD3E* (2)  *RAG1* (1)  *IL2RG* (1)  *STK4* (1)  *CD3D* (1)  *DOCK2* (1) |
| T-B-NK+ (84) | *RAG1* (20)  *RAG2* (13)  *DCLRE1C* (12)  *NHEJ1* (1)  *PNP* (1) |
| T-B-NK- (32) | *ADA* (15)  *PNP* (2)  *AK2* (1)  *IL2RG* (1)  *RAG1* (1) |
| T+B-NK+ (19) | *RAG1* (3)  *RAG2* (4)  *DCLRE1C* (1)  *NHEJ1* (1)  *STIM1* (1) |
| T+B-NK- (4) | *IL2RG* (2)  *ADA* (2) |
| T+B+NK- (7) | *IL2RG* (2)  *JAK3* (3)  *PRKDC* (1) |
| T+B+NK+ (3) | *CD3D* (1)  *RAG1* (1) |
