# Supplementary material for: Clinical, Immunological, and Molecular Features of Severe Combined Immune Deficiency: A Multi-Institutional Experience From India
Source: Front Immunol. 2021 Feb 8;11:619146. doi: 10.3389/fimmu.2020.619146 (PMC7897653; doi:10.3389/fimmu.2020.619146)
Supplement: Supplementary file 3 [file Table_3.docx]

**Supplementary table 3. Analysis of novel missense mutations in present study**

| **Pt.** | **Gene** | **Type of mutation** | **cDNA** | **AA change** | **Clinical and immunological features** | **Population frequency** | **Prediction of novel missense mutations effect on**  **protein function** | | | |
| --- | --- | --- | --- | --- | --- | --- | --- | --- | --- | --- |
|  |  |  |  |  |  |  | **CADD** (**GRCh38-v1.6)** | **PolyPhen-2** | **PROVEAN** |  |
| Pt. 3 | *IL2RG* | Hemizygous | c.515T>G | p.L172R | This male child was born to non-consanguineously married couple; family history: 3 elder male siblings died at early infancy due to fulminant infections; had presented with recurrent episodes of pneumonia and secondary HLH. Blood counts showed persistent lymphopenia. Immunological investigations are suggestive of T-B+NK- SCID (CD3- 0%, CD19- 86% (808), CD56- 0.3%. Autopsy has shown disseminated BCGosis, angioinvasive aspergillosis, and paucity of thymus and lymphoid tissues suggestive of SCID. Underwent Sanger sequencing of *IL2RG* and *JAK3* genes and only molecular variant identified in this child was in *IL2RG* and mother was carrier for the same variant. | 0 | **26.6. (Chromosome X, Position 71110235)** | 1.000 (Probably damaging) | -5.277 (Cut off=-2.5) Deleterious |  |
| Pt. 6 | *IL2RG* | Hemizygous | c.185G>A | p.C62Y | This male child had a strong X-linked family history. Born of non-consanguineously married couple; 7 maternal uncles (males) had died at early infancy due to opportunistic infections. This child also presented in year 2011 with opportunistic infections- persistent pneumonia, oral thrush, and absent BCG scar. Child also succumbed at early infancy due to severe infection. Immunological investigations are suggestive of T-B+NK- SCID (CD3- 0%, CD19- 96.75% (2,245), CD56- 3.2% (74). Underwent Sanger sequencing of *IL2RG* and *JAK3* genes and only molecular variant identified in this child was in *IL2RG* and mother was carrier for the same variant. A similar missense variant affecting the same amino acid residue has been previously reported as pathogenic c.184T>A, p.C62S (Ref: https://www.ncbi.nlm.nih.gov/clinvar/variation/636917/). | 0 | **25.7.(Chromosome X, Position 71110981)** | 1.000 (Probably damaging) | -9.457 (Cut off=-2.5) Deleterious |  |
| Pt. 35 | *IL2RG* | Hemizygous | c.170T>A | p.L57H | This male child born out of non-consanguineous marriage had a history of opportunistic infections. Blood investigations were suggestive of persistent lymphopenia and Immunological investigations are suggestive of T-B+NK- SCID (CD3- 3% (26), CD19- 94% (818), CD56- 0.4% (3)). CD132 (IL2RG) expression by flow cytometry is also reduced (mentioned in table 3). Child also succumbed at early infancy due to severe infection. Only molecular variant identified in the child was *IL2RG* c.170T>A (analysis done by gene panel that covers *ADA, JAK3, IL2RG, IL7RA, RAG1, RAG2, LIG4* genes) with mother being carrier for the same variant. | 0 | **25.5. (Chromosome X, Position 71110996)** | 1.000 (Probably damaging) | -2.054 (Cut off=-2.5) Neutral |  |
| Pt. 152 | *IL2RG* | Hemizygous | c.520T>A | p.W174R | This male child born of non-consanguineously married couple had several opportunistic infections since early infancy and fungal septicemia (Blood culture: *Candida* sp.). Immunological phenotype was suggestive of T-B+NK- SCID (CD3-0%, CD19-93% (1015), CD56- 2% (23). T-cell proliferation or STAT5 phopshorylation in activated T cells could not be done due to absent T cells.  Sanger sequencing for *IL7RA, JAK3,* and *IL2RG* was done and the only variant identified was a hemizygous variant in *IL2RG* c.520T>A. Mother was identified to be carrier for the same variant.  The child also underwent a successful HSCT and achieved successful engraftment. Child was doing well after HSCT and under follow-up for last 7 years. | 0 | **27.4. (Chromosome X, Position 71110230)** | 1.000 (Probably damaging) | -12.857(Cut off=-2.5) Deleterious |  |
| Pt. 93 | *RAG2* | Homozygous | c.95G>A | p.G32E | This was a female infant born of second-degree consanguineous marriage. Had severe opportunistic infections (disseminated CMV with CMV retinitis) and also succumbed to severe infections at 6^th^ month of life. Chest X ray showed absent thymus. Immunological investigations are suggestive of T-B-NK+ SCID (CD3- 7.6% (167), CD19- 1% (22), CD56- 40% (880)). Only molecular defect identified in this child was *RAG2* c.95G>A (homozygous) (analysis done by NGS and genes covered in this panel is provided in the link <https://www.frontiersin.org/files/Articles/431764/fimmu-10-00023-HTML/image_m/fimmu-10-00023-t008.jpg> (7)) and parents are heterozygous carriers for the same variant. | 0 | **25.8. (Chromosome 11, Position 36594074)** | 1.000 (Probably damaging) | -1.722 (Cut off=-2.5) Neutral) |  |
| Pt. 96 | *RAG2* | Homozygous | c.608G>A | p.G203E | This was a female infant born of second-degree consanguineous marriage. Had recurrent episodes of oral thrush in early infancy and succumbed to severe pneumonia at 3^rd^ month. Blood counts showed persistent lymphopenia and chest X ray showed absent thymus. Immunological investigations are suggestive of T-B-NK+ SCID (CD3- 5% (36), CD19- 7% (50), CD56- 53% (382)). Only molecular defect identified in this child was *RAG2* c.608G>A (homozygous) (analysis done by NGS and genes covered in this panel is provided in the link <https://www.frontiersin.org/files/Articles/431764/fimmu-10-00023-HTML/image_m/fimmu-10-00023-t008.jpg> (7)) and parents are heterozygous carriers for the same variant. | 0 | **25.6. (Chromosome 11, Position 36593561)** | 1.000 (Probably damaging) | -7.495 (Cut off=-2.5) Deleterious |  |
| Pt. 207 | *RAG2* | Homozygous | c.329T>C | p.M110T | This was a female infant born of third-degree consanguineous marriage. Had recurrent episodes of oral thrush and pneumonia in early infancy. Also developed enteroviral meningoencephalitis. Blood counts showed persistent lymphopenia and chest X ray showed absent thymus. Immunological investigations are suggestive of T-B-NK+ SCID (CD3- 28% (109), CD19- 1% (4), CD56- 68% (265)). Naïve T cells are reduced (Naïve Tc – 19%, Naïve Th – 6%).  Only molecular defect identified in this child was *RAG2* c.329T>C (homozygous) (analysis done by NGS and genes covered in this panel are provided in the link <https://www.frontiersin.org/files/Articles/431764/fimmu-10-00023-HTML/image_m/fimmu-10-00023-t008.jpg> (7)) and parents are heterozygous carriers for the same variant. | 0 | **22.9. (Chromosome 11, Position 36593840)** | 0.183  (Benign) | -3.756 (Cut off=-2.5) Deleterious) |  |
| Pt. 209 | *RAG2* | Compound heterozygous | c.303T>G; c.171delG | p.N101K (Novel);  p.K58SfsX73 (previously reported) | This is a female infant who presented with recurrent opportunistic infections (recurrent pneumonia, oral thrush, diarrhoea) and chest wall abscess. Chest X ray showed absent thymus. Bronchoalveolar lavage showed positivity for *Pneumocystis jirovecii*. Blood counts showed persistent lymphopenia. Flow cytometry for lymphocyte immunophenotyping showed T-B-NK+ SCID (CD3- 1.3% (13), CD19- 0, CD56- 60% (581)). Only molecular defect identified in this child was in RAG2 (compound heterozygous, c.329T>C, c.171delG) (analysis done by NGS and genes covered in this panel are provided in the link <https://www.frontiersin.org/files/Articles/431764/fimmu-10-00023-HTML/image_m/fimmu-10-00023-t008.jpg> (7)) and parents are heterozygous carriers for the each one of the variants. Child underwent successful haploidentical HSCT in December 2019 and is doing well. | 0 | **23.6. (Chromosome 11, Position 36593866);** | 1.000 (Probably damaging) | -5.226 (Cut off=-2.5) Deleterious) |  |
| Pt. 22 | *ADA* | Compound heterozygous | c.301C>T  c.461G>T | p.R101W (previously reported)  p.C154F (Novel) | Presented with classical T-B-NK- SCID. ADA levels are low (0.1). % dAXP is elevated (63.9). a Human ADA cDNA carrying the C154F substitution was constructed and found that when it is expressed in an ADA deletion strain of *E. coli*, it yielded 0.03% of WT ADA activity, indicating that the variant has a very deleterious effect. | 0 | **27.9. (Chromosome 20, Position 44625586,)** | 0.999  (Probably damaging) | -8.375 (Cut off=-2.5) Deleterious) |  |
| Pt. 36 | *ADA* | Homozygous | c.407G>A | p.G136D | Presented with classical T+B-NK- SCID (Omenn syndrome). ADA levels are low (0). % dAXP is elevated (21.1). | 0 | **25.7. (Request: Chromosome 20, Position 44625640,)** | 1.000 (Probably damaging) | -6.145 (Cut off=-2.5) Deleterious) |  |
| Pt. 29 | *JAK3* | Compound heterozygous | c.1048C>T;  c.704T>C | p.R350W;  p.M235T | This is a female infant who presented with opportunistic infections (severe pneumonia and recurrent diarrhea, failure to thrive, disseminated CMV with CMV retinits). Immunological phenotype was suggestive of T-B+NK- SCID (CD3- 11.3% (149), CD19- 69.8% (921), CD56- 1.75% (23)). CD132 expression and CD127 expression by flow cytometry was normal. Child underwent a targeted exome analysis. Genes covered in the panel are provided in the link <https://www.frontiersin.org/files/Articles/431764/fimmu-10-00023-HTML/image_m/fimmu-10-00023-t008.jpg> (7).  Only molecular defects identified were the compound heterozygous variants in *JAK3* (c.1048C>T, c.704T>C). Each parent was identified to be the heterozygous carriers for each one of the identified variants (Mother: c.1048C>T; Father: c.704T>C) | 0 | **32. (Chromosome 19, Position 17841483);**  **24.1. (Chromosome 19, Position 17842473)** | 1.000 (Probably damaging);  0.004 (Benign) | -7.136 (Cut off=-2.5) Deleterious);  -2.420 (Cut off=-2.5) Neutral |  |
| Pt. 50 | *JAK3* | Compound heterozygous | c.115delC;  c.T1289C | p.Q39SfsX108;  p.I430T | This is a male infant who presented with opportunistic infections since early infancy. Blood counts showed persistent lymphopenia and immunological phenotype was suggestive of T-B+NK- SCID (CD3- 20% (80), CD19- 73% (292), CD56- 1.4% (5-6). Child succumbed to severe pneumonia at 6^th^ month of age. CD132 expression by flow cytometry was normal. TREC levels by RT-PCR were undetectable.  Whole exome sequencing identified the compound heterozygous variants in *JAK3* c.115delC, c.T1289C and no other pathogenic variants have been observed in other SCID genes. Sanger sequencing confirmed the variants and each parent was identified to be the heterozygous carriers for each one of the identified variants (Mother: c.1289T>C; Father: c.115delC) | 0 | **25.3. (Chromosome 19, Position 17839629)** | 1.000 (Probably damaging); | -4.020 (Cut off=-2.5) Deleterious); |  |
| Pt. 198 | *JAK3* | Homozygous | c.1765G>T | p.G589C | This is a female infant who presented with severe infections - recurrent pneumonia and failure to thrive. Immunological investigations show a T-B+NK- phenotype (CD3- 0.5% (22), CD19- 87.3% (3839), CD56- 2% (88)). CD4 and CD8 T cells are also reduced. STAT5 phosphorylation in activated T cells could not be done due to undetectable T cells. However, CD127 expression in B cells was normal. TREC levels by RT-PCR was very low (1.295). Child underwent a targeted exome analysis. Genes covered in the panel is provided in the link <https://www.frontiersin.org/files/Articles/431764/fimmu-10-00023-HTML/image_m/fimmu-10-00023-t008.jpg> (7).  Only molecular identified was a homozygous variant in *JAK3* c.1765G>T and parents are found to be heterozygous carriers for the same mutation. | 0 | **28.4. (Chromosome 19, Position 17837150)** | 1.000 (Probably damaging) | -8.288 (Cut off=-2.5) Deleterious); |  |
| Pt. 114 | *IL7RA* | Homozygous | c.509G>C | p.R170P | This is a male infant who was born of third-degree consanguineous marriage who was symptomatic since early infancy in form of recurrent pneumonia. He was identified to have disseminated BCG infection. Skin biopsy and gastric lavage demonstrated BCG infection. Blood counts showed persistent lymphopenia. Chest X ray showed absent thymus. Immunological investigations suggestive of T-B+NK+ SCID (CD3- 0%, CD19- 61% (1,007), CD56- 38% (627)). Child underwent a targeted exome analysis. Genes covered in the panel is provided in the link <https://www.frontiersin.org/files/Articles/431764/fimmu-10-00023-HTML/image_m/fimmu-10-00023-t008.jpg> (7).  Only molecular variant identified was a homozygous variant in *IL7RA* c.509G>C and parents are found to be heterozygous carriers for the same mutation. | 0 | **25.4. (Chromosome 5, Position 35871185)** | 0.999  (Probably damaging) | -4.098 (Cut off=-2.5) Deleterious) |  |
| Pt. 200 | *IL7RA* | Homozygous | c.324T>G | p.C108W | This is a male infant who presented with opportunistic infections- recurrent diarrhea, pneumonia, and failure to thrive. Immunological investigations are suggestive of T-B+NK+ SCID (CD3- 0%, CD19- 64% (443), CD56- 31% (214)). Child underwent a targeted exome analysis. Genes covered in the panel is provided in the link <https://www.frontiersin.org/files/Articles/431764/fimmu-10-00023-HTML/image_m/fimmu-10-00023-t008.jpg> (7).  Only molecular variant identified was a homozygous variant in *IL7RA* c.324T>G and parents are found to be heterozygous carriers for the same mutation. | 0 | **23.2. (Chromosome 5, Position 35867408)** | 0.998  (Probably damaging) | -3.459(Cut off=-2.5) Deleterious) |  |
| Pt. 86 | *STIM1* | Homozygous | c.1285C>T | p.R429C | This male infant born to non-consanguineously married parents, had a characteristic clinical phenotype for *STIM1* defect- developmental delay, hypotonia, failure to thrive, recurrent severe pneumonia and diarrhea since early infancy. Elder female sibling died at 7 months of age with severe pneumonia. He also had eczematoid rash, and high IgE levels. Child also had evidence of myopathy in form of generalised hypotonia, sluggish deep tendon reflexes, and raised creatinine phosphokinase levels (300 U/L, Normal: <140). Naïve CD4 and CD8 T cells are reduced for the child (CD3-92.20% (13,683), CD19-2.85% (416), CD56-3.21% (475), CD4+45RA+ - 12.17% compared to 56% in control, CD8+45RA+ - 18.6% compared to 72% in control).  Lymphocyte proliferation by thymidine uptake assay after stimulation with phytohemagglutinin (5 µg/ml), showed decreased proliferative capacity in the patient as compared to control (Control- Unstimulated mean CPM: 52, Stimulated mean CPM: 10,449; Case- Unstimulated mean CPM: 43, Stimulated mean CPM: 207).  Only molecular variant identified in the exome sequence was *STIM1*, c.1285C>T (homozygous) and both parents being heterozygous carriers for the same variant. | 0 | **30. (Chromosome 11, Position 4083309)** | 1.000 (Probably damaging) | -7.234 (Cut off=-2.5) Deleterious) |  |
